# Non-pharmaceutical interventions for COVID-19 reduced the incidence of infectious diseases: a controlled interrupted time-series study

**DOI:** 10.1186/s40249-023-01066-3

**Published:** 2023-03-09

**Authors:** Wenyi Zhang, Yao Wu, Bo Wen, Yongming Zhang, Yong Wang, Wenwu Yin, Shanhua Sun, Xianyu Wei, Hailong Sun, Zhijie Zhang, Shanshan Li, Yuming Guo

**Affiliations:** 1 Division of Disease Surveillance, Chinese PLA Center for Disease Control and Prevention, Beijing, 100071 China; 2 School of Public Health and Preventive Medicine, Monash University, Level 2, 553 St Kilda Road, Melbourne, VIC 3004 Australia; 3 Department of Respiratory and Critical Care Medicine, China-Japan Friendship Hospital, Beijing, 100029 China; 4 Division of Infectious Diseases, Key Laboratory of Surveillance and Early-warning on Infectious Disease, Chinese Center for Disease Control and Prevention, Beijing, 102206 China; 5 Beijing Center for Disease Prevention and Control, Beijing, 100013 China; 6 Department of Epidemiology and Health Statistics, Fudan University, Shanghai, 200032 China

**Keywords:** Non-pharmaceutical intervention, Infectious diseases, COVID-19, Prevalence

## Abstract

**Background:**

Non-pharmaceutical interventions (NPIs) have been implemented worldwide to suppress the spread of coronavirus disease 2019 (COVID-19). However, few studies have evaluated the effect of NPIs on other infectious diseases and none has assessed the avoided disease burden associated with NPIs. We aimed to assess the effect of NPIs on the incidence of infectious diseases during the COVID-19 pandemic in 2020 and evaluate the health economic benefits related to the reduction in the incidence of infectious diseases.

**Methods:**

Data on 10 notifiable infectious diseases across China during 2010–2020 were extracted from the China Information System for Disease Control and Prevention. A two-stage controlled interrupted time-series design with a quasi-Poisson regression model was used to examine the impact of NPIs on the incidence of infectious diseases. The analysis was first performed at the provincial-level administrative divisions (PLADs) level in China, then the PLAD-specific estimates were pooled using a random-effect meta-analysis.

**Results:**

A total of 61,393,737 cases of 10 infectious diseases were identified. The implementation of NPIs was associated with 5.13 million (95% confidence interval [*CI*] 3.45‒7.42) avoided cases and USD 1.77 billion (95% *CI *1.18‒2.57) avoided hospital expenditures in 2020. There were 4.52 million (95% *CI* 3.00‒6.63) avoided cases for children and adolescents, corresponding to 88.2% of total avoided cases. The top leading cause of avoided burden attributable to NPIs was influenza [avoided percentage (AP): 89.3%; 95%* CI* 84.5‒92.6]. Socioeconomic status and population density were effect modifiers.

**Conclusions:**

NPIs for COVID-19 could effectively control the prevalence of infectious diseases, with patterns of risk varying by socioeconomic status. These findings have important implications for informing targeted strategies to prevent infectious diseases.

**Supplementary Information:**

The online version contains supplementary material available at 10.1186/s40249-023-01066-3.

## Background

Following the pandemic of the new coronavirus-severe acute respiratory syndrome coronavirus 2 (SARS-CoV-2), non-pharmaceutical interventions (NPIs) have been widely implemented as part of the coronavirus disease 2019 (COVID-19) response to slow the transmission of the virus. These measures include a set of actions apart from getting a vaccination and taking medicine, such as social distancing, hand hygiene, mask-wearing, stay-at-home orders, and closing schools. Recent studies have shown that NPIs have had a large effect on controlling transmission of SARS-CoV-2 [1]. Variations in disease severity and incidence of COVID-19 across countries could be attributed to different levels of public compliance with NPIs [2, 3]. For example, countries, where masks are required at the first wave of pandemic, showed a lower mortality rate compared with countries against widespread mask-wearing by the public [3].

Infectious diseases have presented a major public health challenge [4, 5]. Despite the huge progress in disease control followed by a decline in mortality from infectious diseases [6], the pandemics of the severe acute respiratory syndrome (SARS), following the emergence of novel influenza A viruses (H5NA, H7N9, and H9N2), Zika virus, Ebola over the past few decades, highlight the substantial effect of infectious diseases on global health [7, 8]. In the past few decades, communicable diseases contributed to a large component of the disease burden, especially for children and adolescents [7, 9, 10]. Globally, it’s estimated that 145 thousand [95% uncertainty interval (*UI*): 99‒200] deaths in 2017 could be attributable to lower respiratory tract infections, with children and the elderly being the major contributors [10]. In many regions of the world, the observed increasing hospitalization rate for infectious diseases further emphasizes the need for cost-effective strategies against infectious diseases [11, 12].

Given its feature of suppressing the spread of SARS-CoV-2, NPIs may also curb the transmission of other contagious viruses with the same transmission route as SARS-CoV-2. For example, the transmission rate of hand, foot, and mouth disease (HFMD) and tuberculosis (TB) might be significantly reduced by mask-wearing, social distancing, and hand washing. Recent studies found a large and significant reduction in influenza activity during the COVID-19 pandemic [13]. However, whether the reduction is associated with the implementation of NPIs remains largely unknown. Knowing to what extent NPIs are effective in reducing infectious diseases is critical to policymakers, as most NPIs are easy to carry out and more cost-effective than immunization programs and disease surveillance.

To date, there are limited data that describe the impact of NPIs for COVID-19 on infectious disease incidence and related health economic burden. Previous studies examining the difference in the incidence of infectious diseases before and after NPIs implementation have mainly focused on influenza [13, 14], and few have estimated the impacts of NPIs. In this study, we aimed to evaluate the effect of NPIs on the incidence of infectious diseases during the COVID-19 pandemic in 2020 based on the nationwide dataset from 2010 to 2020 in China and calculate the health economic benefits related to the reduction in the incidence of infectious diseases.

## Methods

### Study design

A two-stage controlled interrupted time-series (CITS) design was applied to evaluate the association between NPIs during the COVID-19 pandemic in 2020 and the incidence of infectious diseases in China.

### Data sources

#### Infectious diseases data

Data on 10 notifiable infectious diseases (seasonal influenza, TB, measles, scarlet fever, mumps, rubella, varicella, bacillary dysentery, infectious diarrhea, and HFMD) across China during 2010–2020 were extracted from the China Information System for Disease Control and Prevention (CISDCP). All cases were diagnosed by medical staff with a clinical diagnosis and laboratory tests based on national uniform standards [7]. Data were aggregated by provincial-level administrative divisions (PLADs) in sex- and age-specific (0–4 years, 5–19 years, 20–24 years, …, ≥ 80 years) monthly time series.

#### Non-pharmaceutical interventions data

In China, almost all PLADs started the NPIs (known as the public health emergency response in China) at the end of January 2020 to cope with COVID-19. NPIs include a series of measures, such as locked-down cities, canceled flights, closed public facilities, restricting residents from going out in public, and obliging citizens to wear masks. NPIs are classified into four levels (I, II, III, IV), with the severity decreasing from Level I to Level IV. Under the Level I response, the most intense NPIs were initiated, including stay-at-home orders, closure of nonessential businesses, restaurants, and hotels, prohibition on gatherings, and postponement of the reopening of schools and colleges. The Level II response marks the period during which normal work and production are allowed while working from home is encouraged. Communities with COVID cases are under closed-off management, banning outsiders from entering and allowing no one to leave. People are required to have their temperature taken, register, and check the health codes from entering. Under the III or IV levels response, educational institutes and businesses reopen, but some major intervention events still remain, e.g., social distancing, wearing masks, and routine temperature monitoring. Timelines of NPIs for each PLAD in 2020 were summarized in Additional file 1: Table S1.

#### Meteorological data

We collected hourly data on ambient temperature, ambient dew point temperature, and total precipitation (at 2 m above the land surface) for the study period from the fifth generation European Centre for Medium-Range Weather Forecasts (ECMWF) atmospheric reanalysis (ERA5) dataset (https://www.ecmwf.int/en/forecasts/datasets/reanalysis-datasets/era5) and transformed them into daily observations by averaging all hourly observations within each day. Daily mean relative humidity was calculated using daily mean temperature and daily mean dew point temperature. Daily data were then aggregated into monthly data. All meteorological data were linked to each PLAD by calculating the average value of all grids overlaying the area.

#### Socioeconomic indexes for areas

We collected three available PLAD-level socioeconomic indicators that potentially affect the prevalence of infectious diseases from the China Statistical Yearbook from 2010 to 2021, including urbanization rate (the proportion of the population living in urban areas), population density, and gross domestic product (GDP) per capita. The 31 PLADs were classified into four quartiles (Qs) groups (Q1–Q4: low, lower middle, higher middle, and high) for each indicator. All GDP data were adjusted to 2020 US dollars according to the Consumer Price Index (CPI) in China and average exchange rate between currencies of China and the United State in 2020.

#### Health care expenditure data

We collected information on per-capita inpatient expenditure from the statistical yearbook of China’s health system. For diseases lack of expenditure data, we screened the literature and extracted data from the latest study in China. All expenditures were adjusted to 2020 US dollars according to the CPI in China and average exchange rate between currencies of China and the United State in 2020 (Additional file 1: Table S2).

### Statistical analysis

#### Two-stage controlled interrupted time-series analysis

**Fig. 1:**
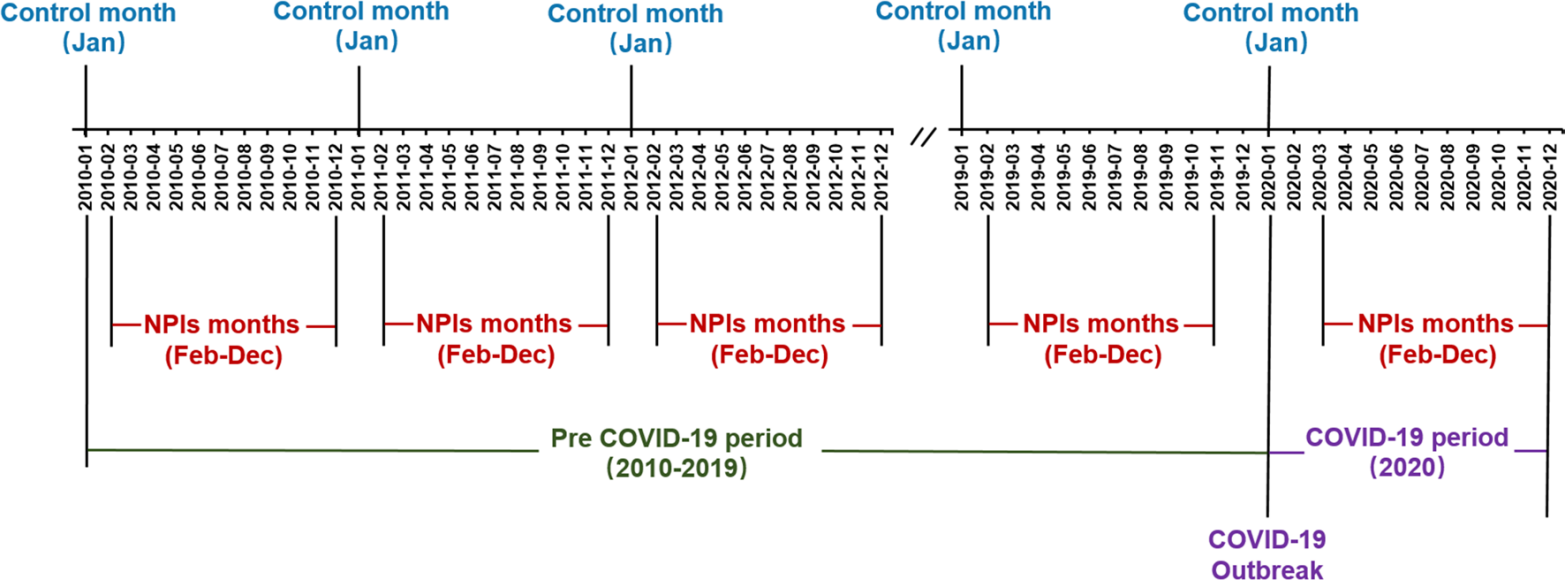
Timelines of COVID-19, non-pharmaceutical interventions during the study period, and the selection of control series

For each infectious disease, a two-stage CITS analysis was applied to quantify the impacts of the NPIs on infectious diseases during the COVID-19 pandemic in 2020 [15]. This design is developed from the basic interrupted time-series (ITS) design that involves a before-after comparison in a single population exposed to the intervention between the observed change in the outcome of interest and the best approximation of the true counterfactual [16, 17]. To provide stronger evidence to support a causal effect of the intervention on the outcome of interest, CITS design includes a control series to exclude problems due to co-interventions or other events occurring around the time of intervention [16]. In this study, we compared the infectious disease incidence before the NPIs with the post-NPIs period. To build a self-controlling design, we selected the first month (January) of each year as the control series as NPIs were not introduced to the study population until the end of January, 2020 (Fig. 1).

In the first stage, a quasi-Poisson time-series regression was applied in each PLAD [18, 19]. The PLAD-specific equation was as follows:

$${Y}_{it}\sim Poisson\left(\mu ;\theta \right)$$

$$\text{E}\left({Y}_{it}\right)={\text{exp}(\alpha }_{i}+{\beta }_{i}NPIs+{{\epsilon }_{i}Group+\gamma }_{i}{Trend}_{secular}{+\delta }_{i}{Trend}_{season}+ns\left({Temp}_{it}\right)+ns\left({RH}_{it}\right)+ns\left({Prep}_{it}\right)+\text{log}\left(POP\right))$$

$$VAR\left({Y}_{it}\right)=\theta \mu$$

where ${Y}_{it}$ denotes cases in PLAD $i$ on month $t$; ${\alpha }_{i}$ is the intercept in PLAD $i$; ${\beta }_{i}$, ${\gamma }_{i}$, and ${\delta }_{i}$ represent the coefficients in PLAD $i$. $NPIs$ is a binary variable to indicate the introduction of NPIs (coded 1) and without the NPIs (coded 0) in the model [20]. $Group$ is a binary variable representing the intervention group (Group = 1, February to December) or control group (Group = 0, January). ${Trend}_{secular}$ denotes a continuous term for time (months since the start of the study) to model the secular trend. ${Trend}_{season}$ stands for an indicator for the month of the year to control for seasonality. $ns\left({Temp}_{it}\right)$, $ns\left({RH}_{it}\right)$, and $ns\left({Prep}_{it}\right)$ are mean temperature, relative humidity, and precipitation controlled in the model using natural spline functions with 3 degrees of freedom (*df* s). Population was also included in the model as an offset term, $\text{l}\text{o}\text{g}\left(POP\right)$. $VAR\left({Y}_{it}\right)$ and $\mu$ denote the variance and expectation of ${Y}_{it}$, and $\theta$ is an over-dispersion parameter. As we only had the 1-year (year 2020) data after the intervention which couldn’t generate the slope of the post-intervention trend, we assumed a constant slope before and after the intervention. The effect of NPIs was expressed as the intercept change (${\beta }_{i}$). As the incidence of infectious diseases had seasonal trends that varied by year, we kept the month constant in different years (using the sixth month for each year) when estimating the secular trend and slope change before and after the intervention. The counterfactual incidence in 2020 was predicted using the same model while we change the intervention term in the model (NPIs) from 1 to 0, assuming that there is no intervention in 2020.

In the second stage, we pooled the PLAD-specific estimates using a random-effect meta-analysis with restricted maximum likelihood estimation [21, 22]. The impacts of NPIs on infectious diseases’ incidence were expressed as the incidence rate ratio (IRR, calculated as the exponents of coefficients) and 95% confidence intervals (95%* CI*).

#### Estimation of avoided cases and hospital expenditures

PLAD-specific number of avoided cases (AC) associated with NPIs during the COVID-19 pandemic in 2020 was calculated using the following equation:

$${AC}_{i}={C}_{i}\times (1-IRR)/IRR$$

where ${C}_{i}$ is the number of observed cases for PLAD $i$ during the COVID-19 pandemic in 2020, $IRR$ is the pooled estimate in the second stage. We calculated the total number of AC by summing all the values of ${AC}_{i}$. The avoided percentage of cases was then calculated at the PLAD level as well as the national level by dividing the total number of AC by the sum of the ${C}_{i}$ and AC. We calculated 95%* CI* of AC (${AC}_{low}$ and ${AC}_{high}$) using the 95%* CI* of IRR. Under the assumption that all cases were admitted to the hospital after being diagnosed, we estimated the avoided hospital expenditures during the COVID-19 pandemic in 2020 as $\text{A}\text{C}\times {cost}_{per capita}$ (${AC}_{low}\times {cost}_{per capita}$ to ${AC}_{high}\times {cost}_{per capita}$), where ${cost}_{per capita}$ denotes the per-capita healthcare expenditure in 2020.

#### Stratification analyses

First, we performed stratified analyses by sex and age group (0–4 years, 5–19 years, and ≥ 20 years). Secondly, we repeated the analyses and pooled the PLAD-specific estimates in different socioeconomic groups (quartiles of urbanization rate, GDP per capita, and population density). Finally, stratified analysis was conducted by different levels of NPIs. Only three levels of NPIs (I, II, and III) were used as few PLADs downgraded their public health response to level IV. We used random effect meta-regression fitted by the maximum likelihood method to compare the effects estimated in different groups [23].

#### Sensitivity analyses

We conducted several sensitivity analyses to check the robustness of our results. Firstly, we repeated the analyses using alternative *df* values (from 3 *df* to 4, 5, and 6 *df*) for mean temperature, relative humidity, and precipitation. Secondly, we used alternative moving average lag structures for all meteorological factors: lag 0–1 (current month and preceding 1 month) and lag 0–2 (current month and preceding 2 months). Thirdly, we refitted the CITS models using data from 2017 to 2020 to avoid inconsistencies in trends between different periods. Finally, for diseases with adequate sample sizes with the elderly, we restricted analyses to people aged between 20 and 54 and people aged above 55, respectively. We used the statistical software R 4.0.1 (Lucent Technologies, Jasmine Mountain, USA) to perform all analyses. A two-sided *P*-value < 0.05 was considered statistically significant.

## Results

**Fig. 2:**
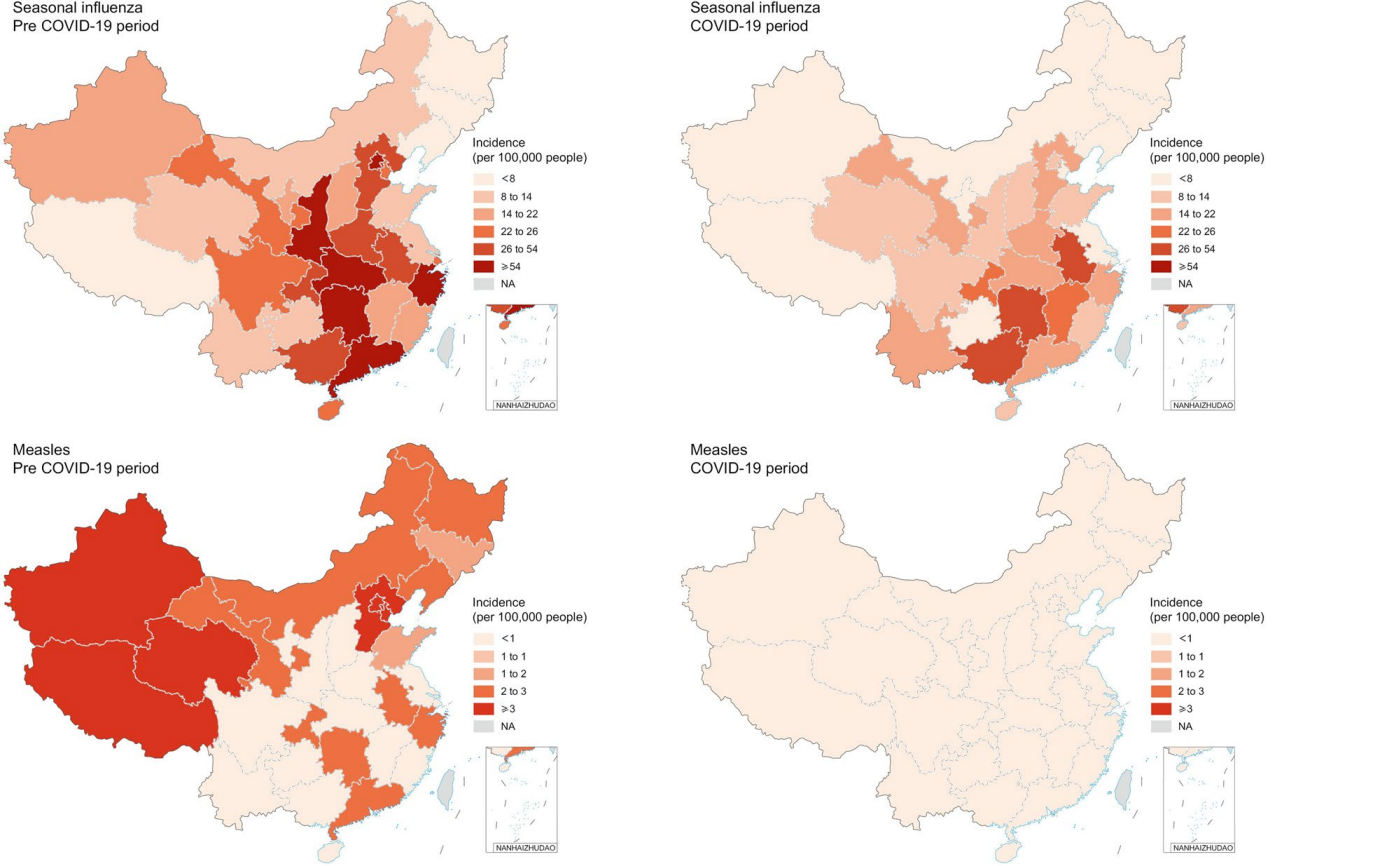
The average monthly incidence of infectious diseases in 31 PLADs of China. Pre COVID-19 period: non-pharmaceutical intervention months (February–December) from 2010–2019; COVID-19 period: non-pharmaceutical intervention months (February–December) in 2020

A total of 61,393,737 cases of ten infectious diseases were identified, among which 59.8% were males. More than two-thirds of cases were children and adolescents. Seasonal influenza, infectious diarrhea, and HFMD had high endemic levels (Additional file 1: Table S3). We observed a relatively high incidence of seasonal influenza and mumps in Middle China. TB, measles, and scarlet fever were more likely to be prevalent in Northwest China, while infectious diarrhea and HFMD showed higher incidence in East and South China (Fig. 2, Additional file 1: Fig. S1). The maps in Fig. 2 and Additional file 1: Fig. S1 showed a reduction in the monthly incidence of all diseases after the introduction of NPIs for most PLADs. A higher reduction in incidence after the implementation of NPIs was observed for seasonal influenza in Beijing and Shaanxi; for varicella in Beijing; for mumps in Chongqing and Hunan; for infectious diarrhea in Beijing; for scarlet fever and bacillary dysentery in Xinjiang; for rubella in Liaoning, Xizang and Chongqing; and for HFMD in Guangdong. Compared to surrounding PLADs, Hubei, the most affected PLAD by COVID-19, showed a higher reduction in the incidence of seasonal influenza and TB (Fig. 2, Additional file 1: Fig. S1).

**Fig. 3:**
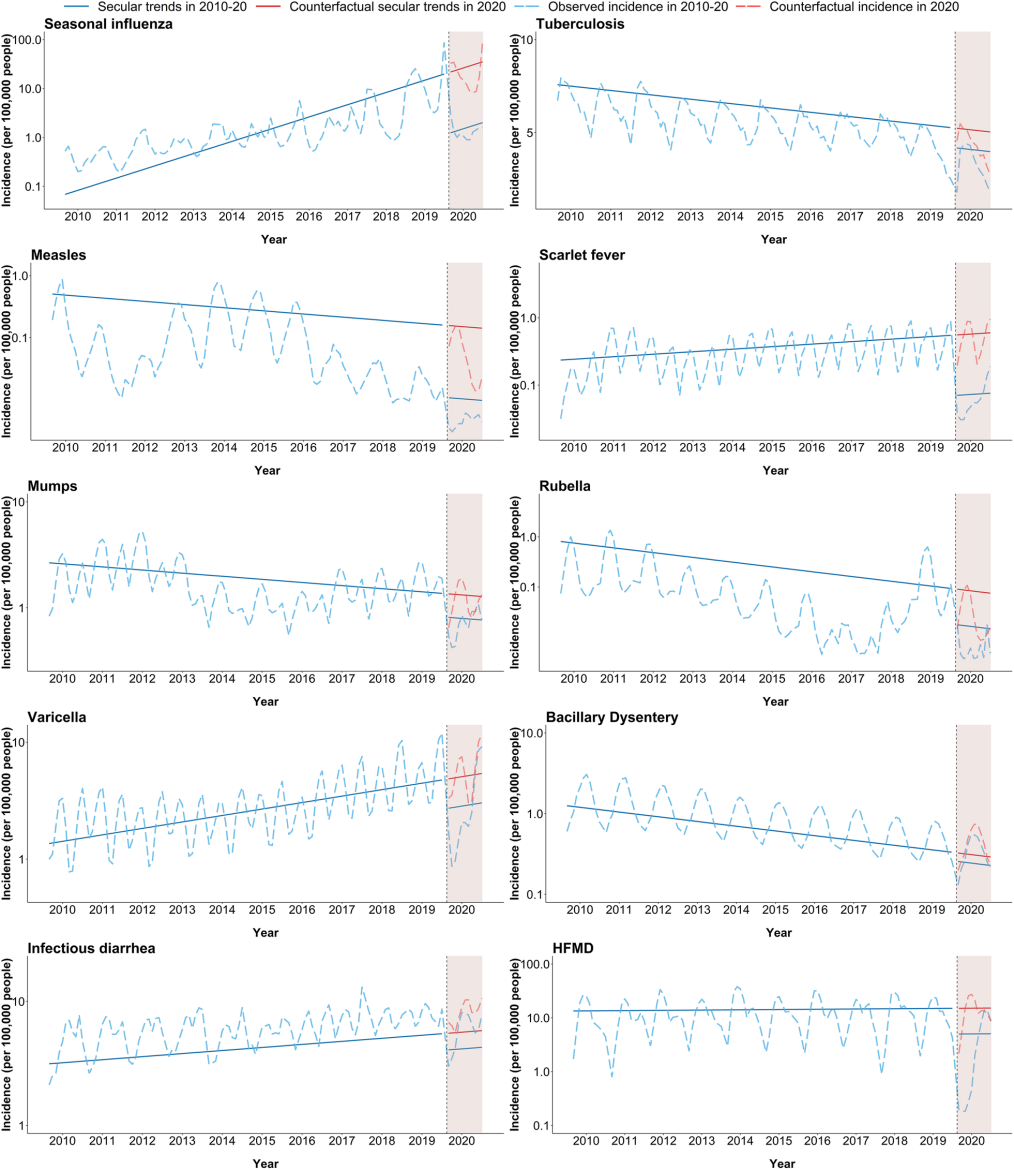
Observed monthly incidence during 2010–20 and predicted incidence without non-pharmaceutical interventions in 2020. Secular trends were estimated using the sixth month of each year to control for seasonality. The effects of non-pharmaceutical interventions were expressed as the slope change before and after the intervention. The number of rates is represented in log scale on the y-axis. Vertical line and shaded areas: introduction of non-pharmaceutical interventions. HFMD: Hand, foot, and mouth disease

Figure 3 shows the monthly incidence rates from 2010 to 2020 and counterfactual prediction of incidence rates in 2020 without NPIs. Compared to counterfactual prediction of incidence in 2020, a reduction in observed incidence in 2020 is shown for most diseases, especially seasonal influenza, measles, and scarlet fever. The corresponding box plots were shown in Additional file 1: Fig. S2.

### Associations between NPIs and infectious diseases’ incidence

**Fig. 4:**
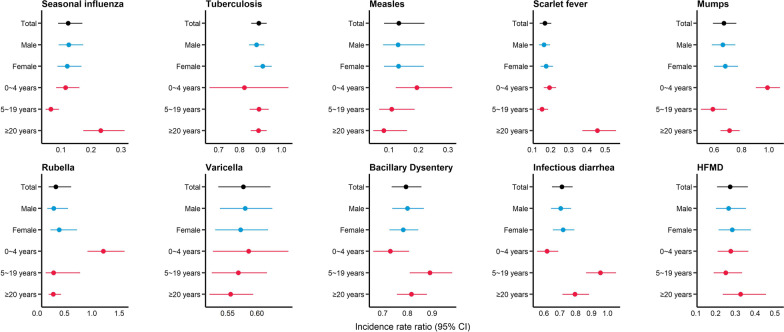
Pooled incidence rate ratio of infectious disease incidence associated with non-pharmaceutical interventions. The exact values can be found in Table S4. HFMD: Hand, foot, and mouth disease

There was a significant reduction in the incidence of all diseases after the NPIs implementation compared to counterfactual prediction of incidence in 2020 (exact values were shown in Additional file 1: Table S4). We estimated an 89% (IRR: 0.11, 95%* CI* 0.07‒0.15) decrease in the incidence rate of seasonal influenza compared to counterfactual scenario, which was the highest among all diseases, followed by measles (88%; IRR: 0.12, 95%* CI* 0.07‒0.20) and scarlet fever (86%; IRR: 0.14, 95%* CI* 0.11‒0.18). TB had the lowest but still significant reduction in incidence after NPIs implementation, with an IRR of 0.87 (95%* CI* 0.83‒0.91) (Fig. 4, Additional file 1: Table S4).

The reduction in incidence after the NPIs implementation varied by age group (Fig. 4). The effect of NPIs was more pronounced among children aged 5–19 years with scarlet fever and mumps; while less pronounced among children of the same age with infectious diarrhea. A larger decrease in the incidence of rubella associated with NPIs was found among those aged above 20 compared to those aged below 20 years.

### Avoided cases and economic burden due to NPIs

**Fig. 5:**
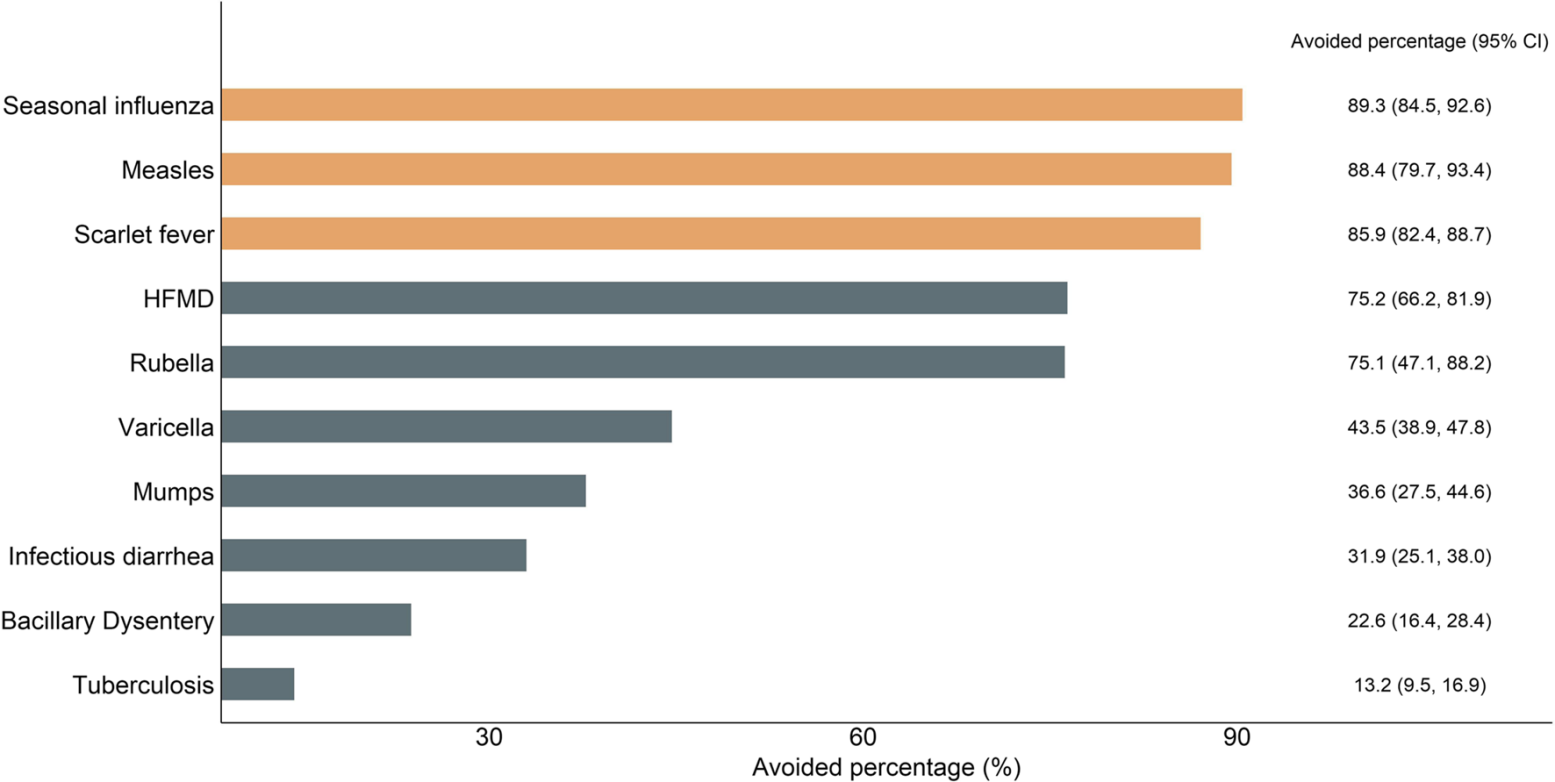
Percentage of cases avoided associated with non-pharmaceutical interventions during the COVID-19 pandemic in 2020. HFMD: Hand, foot, and mouth disease. Orange bars represent the top three diseases with the highest percentage of cases avoided

Figure 5 shows the avoided disease burden due to NPIs during the COVID-19 pandemic in 2020. There were 5.13 million (95%* CI* 3.45‒7.42) avoided cases associated with NPIs for ten infectious diseases during the COVID-19 pandemic in 2020, including 2.24 million HFMD cases, accounting for 75.2% (95%* CI* 66.2‒81.9) of total HFMD cases; 1.81 million seasonal influenza cases, accounting for 89.3% (95%* CI* 84.5‒92.6) of total seasonal influenza cases; 0.44 million infectious diarrhea cases, accounting for 31.9% (95%* CI* 25.1‒38.0) of total infectious diarrhea cases. Rubella had the lowest avoided cases (3318 cases, 95%* CI* 981‒8276) due to NPIs, accounting for 75.1% (95%* CI* 47.1‒88.2) of total rubella cases (Fig. 5; Table 1). The age- and sex-stratified percentages of cases avoided were shown in Additional file 1: Figs. S3, S4.

**Table 1:** Number of cases and health care expenditures avoided associated with non-pharmaceutical interventions

Diseases	Total	Male	Female	0‒4 years	5‒19 years	≥ 20 years
Number of cases avoided‒thousand (95%* CI*)						
Seasonal influenza	1807 (1185‒2705)	951 (626‒1419)	856 (558‒1289)	802 (521‒1214)	738 (467‒1153)	327 (215‒480)
Tuberculosis	93 (64‒124)	70 (50‒91)	25 (15‒35)	0 (-0‒0)	6 (4‒9)	87 (59‒117)
Measles	5 (3‒10)	3 (2‒6)	2 (1‒4)	2 (1‒4)	1 (0‒1)	2 (1‒4)
Scarlet fever	66 (51‒85)	40 (31‒51)	27 (20‒35)	20 (16‒26)	46 (35‒60)	0 (0‒1)
Mumps	65 (43‒91)	39 (26‒54)	26 (17‒37)	2 (-1‒5)	54 (35‒77)	7 (5‒9)
Rubella	3 (1‒8)	2 (1‒6)	1 (0‒3)	0 (0‒0)	2 (0‒7)	1 (1‒2)
Varicella	399 (330‒475)	211 (174‒250)	190 (156‒226)	65 (49‒83)	266 (217‒318)	83 (71‒96)
Bacillary Dysentery	16 (11‒21)	8 (5‒11)	8 (5‒11)	6 (4‒8)	1 (0‒2)	8 (5‒11)
Infectious diarrhea	441 (316‒578)	246 (179‒319)	197 (138‒262)	293 (217‒378)	9 (-2‒22)	128 (72‒190)
HFMD	2235 (1441‒3320)	1309 (850‒1935)	923 (590‒1377)	1978 (1285‒2917)	232 (151‒343)	14 (8‒22)
Health care expenditures avoided‒million USD (95%* CI*)						
Seasonal influenza	826 (542‒1237)	435 (286‒649)	391 (255‒589)	367 (238‒555)	337 (214‒527)	149 (98‒219)
Tuberculosis	138 (94‒183)	103 (73‒134)	36 (22‒52)	0 (− 0‒0)	10 (6‒13)	129 (87‒172)
Measles	2 (1‒4)	1 (1‒3)	1 (1‒2)	1 (1‒2)	0 (0‒1)	1 (0‒2)
Scarlet fever	24 (18‒31)	14 (11‒18)	10 (7‒12)	7 (6‒9)	17 (13‒22)	0 (0‒0)
Mumps	21 (14‒30)	13 (8‒18)	9 (6‒12)	1 (-0‒2)	18 (11‒25)	2 (2‒3)
Rubella	0 (0‒1)	0 (0‒0)	0 (0‒0)	-0 (-0‒0)	0 (0‒0)	0 (0‒0)
Varicella	68 (56‒80)	36 (29‒42)	32 (26‒38)	11 (8‒14)	45 (37‒54)	14 (12‒16)
Bacillary Dysentery	8 (6‒11)	4 (3‒6)	4 (3‒6)	3 (2‒4)	1 (0‒1)	4 (3‒6)
Infectious diarrhea	103 (74‒135)	58 (42‒75)	46 (32‒61)	69 (51‒89)	2 (-1‒5)	30 (17‒44)
HFMD	580 (374‒861)	340 (220‒502)	239 (153‒357)	513 (333‒757)	60 (39‒89)	4 (2‒6)

HFMD: Hand, foot, and mouth disease; CI: Confidential interval; USD: United States Dollars

The reduction in incidence translates into a considerable economic benefit due to NPIs, avoiding USD 1.77 billion (95%* CI* 1.18‒2.57) hospital expenses in total. The highest avoided hospital expenses were observed for seasonal influenza (USD 826 million, 95%* CI* 542‒1237), followed by HFMD (USD 580 million, 95%* CI* 374‒861), TB (USD 138 million, 95%* CI* 94‒183), and infectious diarrhea (USD 103 million, 95%* CI* 74‒135) (Table 1).

### The modification effect of socio-economic status and levels of NPIs

**Fig. 6:**
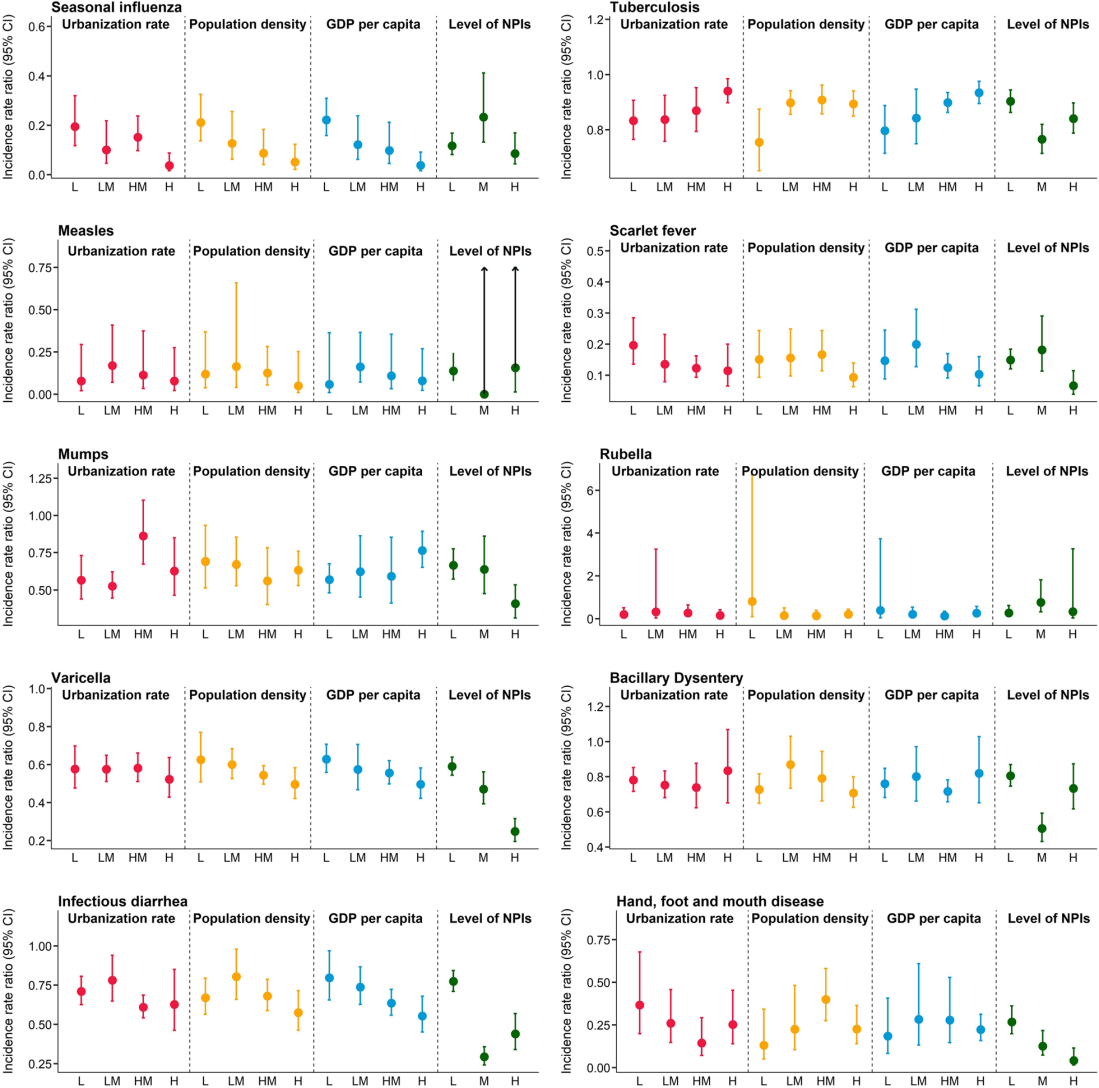
Pooled incidence rate ratio associated with non-pharmaceutical interventions stratified by socioeconomic and demographic indicators. The exact values can be found in Additional file 1: Tables S5–S8. L: Low; LM: Lower middle; HM: Higher middle; H: High

Urbanization rate and population density are shown to significantly modify the incidence of seasonal influenza associated with NPIs. The lower the urbanization rate and population density levels, the higher effect of NPIs (Additional file 1: Tables S5, S6). As shown in Fig. 6, GDP per capita was identified as a significant modifier for most diseases, with a lower effect of NPIs in less-developed PLADs for seasonal influenza, varicella, and infectious diarrhea, but a higher effect of NPIs in less-developed PLADs for TB and mumps (Additional file 1: Table S7). PLAD-specific effect estimates and GDP per capita level are shown in Additional file 1: Table S8. Stratification analyses by different levels of NPIs showed that even the lowest level of NPIs could lead to a significant reduction in incidence for all infectious diseases (Fig. 6, Additional file 1: Table S9). For diseases like varicella, infectious diarrhea, and HFMD, the middle- and high-level NPIs could lead to a significantly higher reduction in incidence compared to low-level NPIs (Additional file 1: Table S9).

### Sensitivity analyses

Analyses of changes in model parameters and study periods show similar results to main analyses (Additional file 1: Tables S10, S11, S12, S13, S14 and Fig. S5). When we restricted the analysis to people aged between 20 and 54 and people aged > 55, weaker effects of NPIs were identified with the increase in age (Additional file 1: Table S15).

## Discussion

The introduction of NPIs during the COVID-19 pandemic in 2020 led to a significant decrease in the incidence of infectious diseases, particularly seasonal influenza, measles, and scarlet fever. The impacts of NPIs could be significantly modified by demographical and socioeconomic indicators. Considerable healthcare expenditures were avoided with the adoption of NPIs.

Our study showed that the implementation of NPIs could contribute to the reduction of the incidence of a set of infectious diseases with different transmission routes, such as seasonal influenza and infectious diarrhea. It has been proven to be effective in reducing the transmission of directly transmitted respiratory infections by applying NPIs [24, 25], which may take effect through directly cutting off the transmission route of respiratory pathogens. Besides, infectious diseases with other transmission modes could also be reduced by applying NPIs. It was observed that the incidence of norovirus outbreaks in nine US states showed a dramatic decline in April 2020 [26]. Similarly, we also observed that over 30% of cases of infectious diarrheal were avoided in 2020 under the implication of NPIs. Thus, these findings imply that NPIs could reduce the incidence of infectious diseases by reducing human-to-human contact, frequent surface disinfection, and enhanced hand hygiene [26, 27].

The economic burden related to infectious diseases should not be neglected. Globally, it was projected that annual economic losses from the influenza pandemic would be about USD 500 billion, corresponding to 0.6% of global income [28]. The COVID-19 pandemic was estimated to threaten nearly 20% of the GDP and reduce the wage income by 16% in the US [29]. In the present study, we found considerable healthcare expenditures could be avoided with the introduction of NPIs. In addition to direct healthcare expenditures, the reduction of infectious diseases due to NPIs could help relieve overburdened healthcare facilities [30]. More importantly, infectious diseases are disproportionately affecting low- and middle-income countries and populations with lower socioeconomic status, which may lead to a cycle of poverty [31]. Our findings from stratification analyses by different levels of NPIs indicate that it’s easy to achieve a satisfying effect with less stringent NPIs (low-level NPIs), simply by social distancing and wearing masks in public. These measures may be easily executed in counties or communities with weak health system infrastructure. High-level NPIs mean enforcing the most stringent actions by the government like travel restrictions, school closures, bans of small gatherings, or even stay-at-home orders. As reported in a recent study, nearly 70% of countries across the globe had compulsory stay-at-home orders at any point in time between the recognition of widespread COVID-19 and the end of April 2020 [32]. Despite their efficacy in controlling the spread of COVID-19, these measures have resulted in an unprecedented economic decline [33], with a higher unemployment rate in many countries [34, 35]. As for endemic infectious diseases, there is a trade-off between economic development and the prevention of diseases. Further cost-benefit analyses for NPIs are warranted.

Children and adolescents remain vulnerable to most infectious diseases, especially respiratory and gastrointestinal infections [7, 36]. A study in China screened 44 notifiable infectious diseases among children and adolescents and identified mumps, seasonal influenza, infectious diarrhea, HFMD, and scarlet fever as the most common infections [7]. Our results showed that there were 4.52 million avoided cases due to NPIs in 2020 for children and adolescents, corresponding to 88.17% of total avoided cases during the COVID-19 pandemic. For children aged below 4, 90% of avoided cases of seasonal influenza and 83% of avoided cases of scarlet fever were associated with NPIs; these figures reached 95% (seasonal influenza) and 88% (scarlet fever) for children aged between 5 and 19 years, respectively. Our findings highlight the effect of NPIs on infectious disease prevention among children and adolescents and suggest that children and adolescents could be the targets of NPIs to prevent infectious diseases.

This study benefits from the application of the CITS design with a control series that allowed us to obtain the effect of NPIs on infectious diseases excluding problems due to co-interventions or other events occurring around the time of NPIs. As the timeline of NPIs and factors affecting the transmission of infectious diseases may vary across PLADs, PLAD-specific analysis after adjustment of long-term trend, seasonality, and meteorological factors offered a fine characterization of the impact of NPIs.

Some limitations should be acknowledged. One limitation of our study concerns the short duration of high-level NPIs. As the COVID-19 pandemic was mostly controlled in China at the beginning of March, the most intense NPIs lasted for only 1 month for most PLADs, which may lead to an underestimation of the effect of high-level NPIs. We should also acknowledge that there might be uncertainties introduced by the change in health services utilization during the COVID-19 pandemic. Some studies observed a reduction in visits to medical centers in early 2020 due to general quarantine conditions or fear of hospital-acquired infections [37, 38]. This might lead to the overestimation of our results. However, given that many infectious diseases share some of the same symptoms with COVID-19, like fever, cough, and severe acute respiratory distress syndrome, detection rates of these diseases should not be largely affected. In some areas of China, residents buying antipyretics, antivirals, and drugs that target coughs and sore throats must undergo COVID-19 testing to root out undetected virus infections. Another study found that the flu detection rate among Chinese children is stable during the COVID-19 pandemic compared to the same period during 2018–19 despite the decrease in pediatric outpatient visits [39].

The analysis was based on the monthly incidence of infectious diseases, so we were unable to apply further analyses on a shorter time scale due to the limitation of the dataset. This may be addressed when more detailed data were obtained. Besides, although all cases were diagnosed by medical staff and confirmed by laboratory tests, there exists the possibility of underestimating the incidence of infectious diseases because some health facilities at the township level in rural areas were not included in the CISDCP [40]. The low number of cases in the elderly prevents the ability to assess the differentiated effect of NPIs on infectious diseases across finer age groups. Finally, we should acknowledge that infections are not always required admission to the hospital, and avoided hospital expenditures could be overestimated.

## Conclusions

Our study indicates that NPIs are an effective way to control the transmission of infectious diseases, with patterns of risk varying by age, socio-economic status, and levels of NPIs. These findings have important implications for informing targeted strategies to prevent infectious diseases.

## Supplementary Information

**Additional file 1.** Additional figures and tables.

## Data Availability

The datasets used and/or analysed during the current study are available from the corresponding author on reasonable request.

## Abbreviations

NPI: Non-pharmaceutical intervention
COVID-19: Coronavirus disease 2019
SARS: Severe acute respiratory syndrome
SARS-CoV-2: Severe acute respiratory syndrome coronavirus 2
HFMD: Hand, foot, and mouth disease
TB: Tuberculosis
UI: Uncertainty interval
CITS: Controlled interrupted time-series
ITS: Interrupted time-series
CISDCP: China Information System for Disease Control and Prevention
ECMWF: European Centre for Medium-Range Weather Forecasts
ERA5: Fifth generation European Centre for Medium-Range Weather Forecasts atmospheric reanalysis
Q: Quartile
GDP: Gross domestic product
CPI: Consumer price index
df: Degrees of freedom
IRR: Incidence rate ratio
CI: Confidence interval
AC: Avoided cases
