# Supplementary material for: Non-pharmaceutical interventions for COVID-19 reduced the incidence of infectious diseases: a controlled interrupted time-series study
Source: Infect Dis Poverty. 2023 Mar 9;12:15. doi: 10.1186/s40249-023-01066-3 (PMC9996566; doi:10.1186/s40249-023-01066-3)
Supplement: Supplementary file 1 — Additional file 1. Additional figures and tables. [file 40249_2023_1066_MOESM1_ESM.docx]

**Non-pharmaceutical interventions for COVID-19 reduced the incidence of infectious diseases: a controlled interrupted time-series study**

**Additional file 1**

**Table of contents**

**Table S1.** The timelines of non-pharmaceutical interventions during the COVID-19 pandemic in 2020, China.

**Table S2.** Per-capita inpatient expenditure for each disease.

**Table S3.** The average monthly number of cases of infectious diseases, in total and stratified by sex and age groups.

**Table S4.** Pooled relative risks of infectious disease incidence associated with non-pharmaceutical interventions during the COVID-19 pandemic.

**Table S5.** Pooled relative risks of infectious disease incidence associated with non-pharmaceutical interventions during the COVID-19 pandemic stratified by urbanization rate level.

**Table S6.** Pooled relative risks of infectious disease incidence associated with non-pharmaceutical interventions during the COVID-19 pandemic stratified by population density level.

**Table S7.** Pooled relative risks of infectious disease incidence associated with non-pharmaceutical interventions during the COVID-19 pandemic stratified by GDP per-capita level.

**Table S8.** Pooled relative risks of infectious disease incidence associated with non-pharmaceutical interventions during the COVID-19 pandemic stratified by non-pharmaceutical interventions level.

**Table S9.** Pooled relative risks of infectious disease incidence associated with non-pharmaceutical interventions during the COVID-19 pandemic, using alternative degrees of freedom for mean temperature.

**Table S10.** Pooled relative risks of infectious disease incidence associated with non-pharmaceutical interventions during the COVID-19 pandemic, using alternative degrees of freedom for mean relative humidity.

**Table S11.** Pooled relative risks of infectious disease incidence associated with non-pharmaceutical interventions during the COVID-19 pandemic, using alternative degrees of freedom for mean precipitation.

**Table S12.** Pooled relative risks of infectious disease incidence associated with non-pharmaceutical interventions during the COVID-19 pandemic, using alternative lag days for meteorological factors.

**Table S13.** Pooled relative risks of infectious disease incidence associated with non-pharmaceutical interventions during the COVID-19 pandemic, using different study periods.

**Table S14.** Pooled relative risks of infectious disease incidence associated with non-pharmaceutical interventions during the COVID-19 pandemic, stratified by age groups.

**Figure S1.** The average monthly incidence of infectious diseases in 31 PLADs of China. Pre COVID-19 period: non-pharmaceutical intervention months (February–December) from 2010–2019; COVID-19 period: non-pharmaceutical intervention months (February–December) in 2020

**Figure S2.** Observed annual incidence rates from 2010 to 2020 (green-colored boxes) and predicted incidence rates without non-pharmaceutical interventions in 2020 using controlled interrupted time series model (purple-colored boxes) for ten infectious diseases.

**Figure S3.** Percentage of cases avoided associated with non-pharmaceutical interventions during COVID-19 pandemic in 2020, stratified by sex. HFMD = hand, foot, and mouth disease. Orange bars represent the top three diseases with the highest percentage of cases avoided.

**Figure S4.** Percentage of cases avoided associated with non-pharmaceutical interventions during COVID-19 pandemic in 2020, stratified by age. HFMD = hand, foot, and mouth disease. Orange bars represent the top three diseases with the highest percentage of cases avoided.

**Figure S5.** Annual incidence rates from 2010 to 2020 (green-colored boxes) and predicted incidence rates without non-pharmaceutical interventions in 2020 using controlled interrupted time series model (purple-colored boxes) for ten infectious diseases. HFMD = Hand, foot, and mouth disease.

**Table S1. The timelines of non-pharmaceutical interventions during the COVID-19 pandemic in 2020, China.**

| PLADs | Levels of non-pharmaceutical interventions (NPIs) | | | |
| --- | --- | --- | --- | --- |
|  | First-level (High) | Second-level  (Middle) | Third-level  (Low) | Fourth-level  (Low) |
| Beijing | 24-Jan | 30-Apr | 6-Jun |  |
|  |  | 16-Jun | 20-Jul |  |
| Tianjin | 24-Jan | 30-Apr | 6-Jun |  |
| Hebei | 24-Jan | 30-Apr | 6-Jun |  |
| Hubei | 24-Jan | 22-Jan | 13-Jun |  |
|  |  | 2-May |  |  |
| Tibet | 30-Jan | 27-Jan |  |  |
|  |  | 7-Mar |  |  |
| Ningxia | 25-Jan | 28-Feb | 6-May |  |
| Shanghai | 24-Jan | 24-Mar | 9-May |  |
| Guangdong | 23-Jan | 24-Feb | 9-May |  |
| Shanxi | 25-Jan | 24-Feb | 10-Mar |  |
| Jiangsu | 24-Jan | 24-Feb | 27-Mar |  |
| Zhejiang | 23-Jan | 2-Mar | 23-Mar |  |
| Anhui | 24-Jan | 25-Feb | 15-Mar |  |
| Liaoning | 25-Jan | — | 22-Feb |  |
| Jiangxi | 24-Jan | 12-Mar | 20-Mar |  |
| Shandong | 24-Jan | 7-Mar | 5-May |  |
| Henan | 25-Jan | 19-Mar | 5-May |  |
| Inner Mongolia | 25-Jan | — | 25-Feb |  |
| Hunan | 23-Jan | 10-Mar | 31-Mar |  |
| Heilongjiang | 25-Jan | 4-Mar | 25-Mar |  |
| Guangxi | 24-Jan | — | 24-Feb |  |
| Hainan | 25-Jan | 24-Jan | 26-Feb |  |
| Chongqing | 24-Jan | 10-Mar | 24-Mar |  |
| Sichuan | 24-Jan | 26-Feb | 25-Mar |  |
| Guizhou | 24-Jan | — | 23-Feb |  |
| Yunnan | 24-Jan | — | 24-Feb |  |
| Jilin | 25-Jan | 26-Feb | 20-Mar |  |
| Shaanxi | 25-Jan | — | 28-Feb |  |
| Gansu | 25-Jan | — | 21-Feb |  |
| Fujian | 24-Jan | 27-Feb | 27-Feb |  |
|  |  | (Middle-risk areas) | (Low-risk areas) |  |
| Xinjiang | 25-Jan | 25-Feb | 7-Mar | 21-Mar |
| Qinghai | 25-Jan | — | 26-Feb | 6-Mar |

**Table S2. Per-capita inpatient expenditure for each disease.**

| Disease | Year | Per-capita inpatient expenditure (currency) | Source |
| --- | --- | --- | --- |
| Seasonal influenza | 2020 | 3200.80 (RMB) | The statistical year book of the China’s health system in 2020 |
| Tuberculosis | 2020 | 10336.13 (RMB) | The statistical year book of the China’s health system in 2020 |
| Measles | 2019 | 442 (USD) | Chen et al [1] |
| Scarlet fever | 2020 | 2510.91 (RMB) | The statistical year book of the China’s health system in 2020 |
| Mumps | 2016 | 2098 (RMB) | Zhao et al [2] |
| Rubella | 2004 | 300.8 (RMB) | Guo et al [3] |
| Varicella | 2017 | 1100 (RMB) | Wang et al [4] |
| Bacillary Dysentery | 2020 | 3646.84 (RMB) | The statistical year book of the China’s health system in 2020 |
| Infectious diarrhea | 2015 | 3525 (RMB) | Tu et al [5] |
| HFMD | 2013 | 1634.20 (USD) | Zheng et al [6] |

HFMD = Hand, foot, and mouth disease. RMB = Renminbi (the Chinese currency). USD = the United States dollar.

**Table S3. The average monthly number of cases of infectious diseases, in total and stratified by sex and age groups.**

| Disease | Subgroup | Number of cases (%) | | | | |
| --- | --- | --- | --- | --- | --- | --- |
|  |  | Control months (January) | | Non-pharmaceutical interventions months  (February-December) | | Total |
|  |  | Pre COVID-19 pandemic | COVID-19 pandemic | Pre COVID-19 pandemic | COVID-19 pandemic |  |
| Seasonal influenza | Total | 106172 (9.7) | 928541 (84.5) | 43875 (4.0) | 19703 (1.8) | 1098291 |
|  | Sex |  |  |  |  |  |
|  | Male | 56376 (9.9) | 480788 (84.1) | 23823 (4.2) | 10631 (1.9) | 571618 |
|  | Female | 49796 (9.5) | 447753 (85.0) | 20052 (3.8) | 9073 (1.7) | 526674 |
|  | Age, years |  |  |  |  |  |
|  | 0-4 | 36125 (10.1) | 299098 (83.9) | 13433 (3.8) | 8009 (2.2) | 356665 |
|  | 5-19 | 34350 (7.9) | 377237 (87.0) | 18372 (4.2) | 3529 (0.8) | 433488 |
|  | ≥20 | 35698 (11.6) | 252206 (81.8) | 12070 (3.9) | 8166 (2.7) | 308140 |
| Tuberculosis | Total | 88866 (31.8) | 59526 (21.3) | 75272 (27.0) | 55546 (19.9) | 279210 |
|  | Sex |  |  |  |  |  |
|  | Male | 61771 (32.0) | 41326 (21.4) | 51977 (26.9) | 37904 (19.6) | 192978 |
|  | Female | 27095 (31.4) | 18200 (21.1) | 23295 (27.0) | 17642 (20.5) | 86232 |
|  | Age, years |  |  |  |  |  |
|  | 0-4 | 108 (32.0) | 63 (18.7) | 113 (33.5) | 53 (15.7) | 337 |
|  | 5-19 | 5139 (29.1) | 3642 (20.7) | 4972 (28.2) | 3879 (22.0) | 17632 |
|  | ≥20 | 83619 (32.0) | 55821 (21.4) | 70187 (26.9) | 51614 (19.8) | 261241 |
| Measles | Total | 1292 (38.7) | 148 (4.4) | 1833 (54.9) | 64 (1.9) | 3337 |
|  | Sex |  |  |  |  |  |
|  | Male | 765 (39.4) | 81 (4.2) | 1058 (54.5) | 36 (1.9) | 1940 |
|  | Female | 527 (37.7) | 67 (4.8) | 775 (55.5) | 28 (2.0) | 1397 |
|  | Age, years |  |  |  |  |  |
|  | 0-4 | 870 (43.2) | 66 (3.3) | 1031 (51.2) | 46 (2.3) | 2013 |
|  | 5-19 | 93 (30.8) | 50 (16.6) | 153 (50.7) | 6 (2.0) | 302 |
|  | ≥20 | 329 (32.2) | 32 (3.1) | 649 (63.5) | 12 (1.2) | 1022 |
| Scarlet fever | Total | 4514 (28.0) | 5748 (35.6) | 4880 (30.3) | 983 (6.1) | 16125 |
|  | Sex |  |  |  |  |  |
|  | Male | 2776 (29.0) | 3250 (33.9) | 2983 (31.2) | 564 (5.9) | 9573 |
|  | Female | 1738 (26.5) | 2498 (38.1) | 1896 (28.9) | 420 (6.4) | 6552 |
|  | Age, years |  |  |  |  |  |
|  | 0-4 | 1320 (27.5) | 1626 (33.9) | 1494 (31.1) | 363 (7.6) | 4803 |
|  | 5-19 | 3148 (28.4) | 4023 (36.2) | 3337 (30.1) | 596 (5.4) | 11104 |
|  | ≥20 | 46 (21.2) | 99 (45.6) | 48 (22.1) | 24 (11.1) | 217 |
| Mumps | Total | 22389 (30.4) | 16539 (22.5) | 24487 (33.2) | 10235 (13.9) | 73650 |
|  | Sex |  |  |  |  |  |
|  | Male | 13653 (30.9) | 9533 (21.6) | 15105 (34.2) | 5936 (13.4) | 44227 |
|  | Female | 8736 (29.7) | 7006 (23.8) | 9381 (31.9) | 4299 (14.6) | 29422 |
|  | Age, years |  |  |  |  |  |
|  | 0-4 | 2828 (23.5) | 1822 (15.1) | 4488 (37.3) | 2893 (24.0) | 12031 |
|  | 5-19 | 17237 (32.3) | 12404 (23.3) | 17610 (33.0) | 6077 (11.4) | 53328 |
|  | ≥20 | 2324 (28.0) | 2313 (27.9) | 2388 (28.8) | 1264 (15.2) | 8289 |
| Rubella | Total | 753 (19.0) | 1098 (27.7) | 2013 (50.8) | 100 (2.5) | 3964 |
|  | Sex |  |  |  |  |  |
|  | Male | 430 (19.2) | 621 (27.7) | 1141 (50.8) | 52 (2.3) | 2244 |
|  | Female | 323 (18.8) | 477 (27.7) | 872 (50.7) | 48 (2.8) | 1720 |
|  | Age, years |  |  |  |  |  |
|  | 0-4 | 183 (32.3) | 35 (6.2) | 324 (57.2) | 24 (4.2) | 566 |
|  | 5-19 | 369 (15.5) | 792 (33.3) | 1174 (49.3) | 45 (1.9) | 2380 |
|  | ≥20 | 201 (19.8) | 271 (26.6) | 514 (50.5) | 31 (3.0) | 1017 |
| Varicella | Total | 53106 (21.7) | 100272 (41.1) | 43734 (17.9) | 47114 (19.3) | 244226 |
|  | Sex |  |  |  |  |  |
|  | Male | 29593 (22.3) | 53708 (40.5) | 24169 (18.2) | 25152 (19.0) | 132622 |
|  | Female | 23514 (21.1) | 46564 (41.7) | 19565 (17.5) | 21962 (19.7) | 111605 |
|  | Age, years |  |  |  |  |  |
|  | 0-4 | 9783 (22.6) | 16486 (38.1) | 9116 (21.1) | 7914 (18.3) | 43299 |
|  | 5-19 | 34971 (22.4) | 63472 (40.7) | 27404 (17.6) | 30250 (19.4) | 156097 |
|  | ≥20 | 8352 (18.6) | 20314 (45.3) | 7214 (16.1) | 8949 (20.0) | 44829 |
| Bacillary Dysentery | Total | 6371 (22.6) | 3212 (11.4) | 13699 (48.6) | 4903 (17.4) | 28185 |
|  | Sex |  |  |  |  |  |
|  | Male | 3582 (23.1) | 1654 (10.7) | 7685 (49.6) | 2579 (16.6) | 15500 |
|  | Female | 2790 (22.0) | 1558 (12.3) | 6014 (47.4) | 2324 (18.3) | 12686 |
|  | Age, years |  |  |  |  |  |
|  | 0-4 | 1939 (22.2) | 875 (10.0) | 4546 (52.1) | 1373 (15.7) | 8733 |
|  | 5-19 | 739 (20.9) | 456 (12.9) | 1710 (48.3) | 635 (17.9) | 3540 |
|  | ≥20 | 3694 (23.2) | 1881 (11.8) | 7443 (46.8) | 2896 (18.2) | 15914 |
| Infectious diarrhea | Total | 89159 (23.5) | 119884 (31.6) | 84691 (22.3) | 85672 (22.6) | 379406 |
|  | Sex |  |  |  |  |  |
|  | Male | 52256 (24.5) | 67072 (31.4) | 48167 (22.5) | 46118 (21.6) | 213613 |
|  | Female | 36902 (22.3) | 52812 (31.9) | 36524 (22.0) | 39554 (23.9) | 165792 |
|  | Age, years |  |  |  |  |  |
|  | 0-4 | 63585 (27.8) | 82832 (36.2) | 44487 (19.4) | 38073 (16.6) | 228977 |
|  | 5-19 | 5191 (16.9) | 8535 (27.8) | 6828 (22.3) | 10111 (33.0) | 30665 |
|  | ≥20 | 20383 (17.0) | 28517 (23.8) | 33376 (27.9) | 37488 (31.3) | 119764 |
| HFMD | Total | 53049 (16.1) | 26104 (7.9) | 184364 (55.8) | 66841 (20.2) | 330358 |
|  | Sex |  |  |  |  |  |
|  | Male | 31513 (16.1) | 14453 (7.4) | 112006 (57.3) | 37590 (19.2) | 195562 |
|  | Female | 21536 (16.0) | 11651 (8.6) | 72358 (53.7) | 29251 (21.7) | 134796 |
|  | Age, years |  |  |  |  |  |
|  | 0-4 | 47657 (16.1) | 22743 (7.7) | 164701 (55.8) | 60171 (20.4) | 295272 |
|  | 5-19 | 5172 (15.6) | 2986 (9.0) | 18856 (56.9) | 6138 (18.5) | 33152 |
|  | ≥20 | 220 (11.4) | 375 (19.4) | 806 (41.7) | 532 (27.5) | 1933 |

HFMD = Hand, foot, and mouth disease.

**
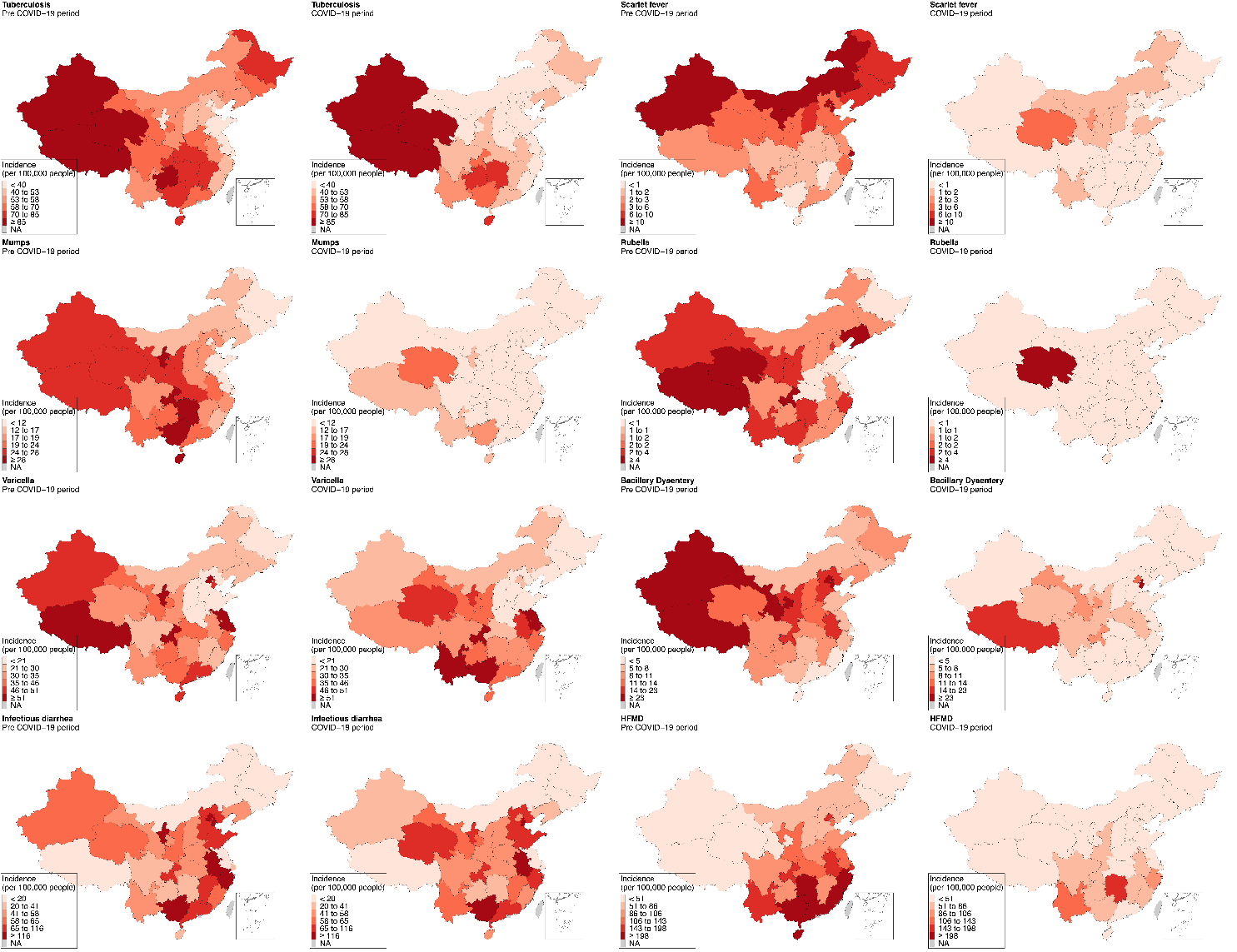
**

**Figure S1. The average monthly incidence of infectious diseases in 31 PLADs of China. Pre COVID-19 period: non-pharmaceutical intervention months (February–December) from 2010–2019; COVID-19 period: non-pharmaceutical intervention months (February–December) in 2020.**

**
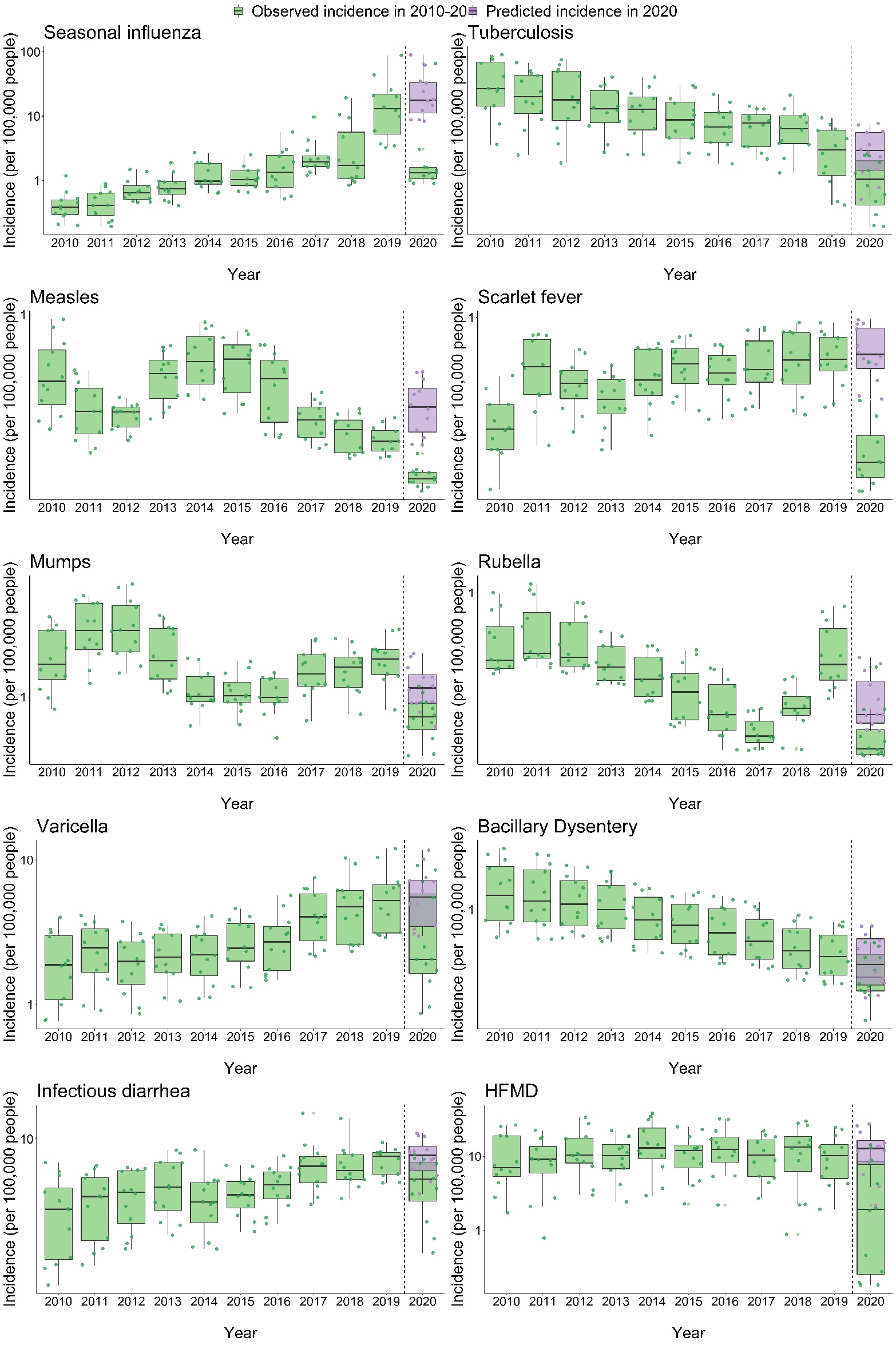
**

**Figure S2. Observed annual incidence rates from 2010 to 2020 (green-colored boxes) and predicted incidence rates without non-pharmaceutical interventions in 2020 using controlled interrupted time series model (purple-colored boxes) for ten infectious diseases.** The number of rates is represented in log scale on the y-axis. HFMD = Hand, foot, and mouth disease. The box shows medians (solid line) and interquartile ranges. The whiskers give the range except for ''outliers'' that are more than 1.5 times the inter-quartile range larger or smaller than the median.

**Table S4. Pooled relative risks of infectious disease incidence associated with non-pharmaceutical interventions during the COVID-19 pandemic.**

| **Diseases** | **Subgroup** | **Relative risk (95% *CI*)** | **P for difference ^*^** |
| --- | --- | --- | --- |
| Seasonal influenza | Total | 0.11 (0.07, 0.15) |  |
|  | Sex |  |  |
|  | Male | 0.11 (0.08, 0.16) | Ref |
|  | Female | 0.10 (0.07, 0.15) | 0.928 |
|  | Age |  |  |
|  | 0-4 years | 0.10 (0.07, 0.14) | Ref |
|  | 5-19 years | 0.05 (0.03, 0.08) | **0.006** |
|  | ≥20 years | 0.22 (0.16, 0.29) | 0.115 |
| Tuberculosis | Total | 0.87 (0.83, 0.91) |  |
|  | Sex |  |  |
|  | Male | 0.86 (0.82, 0.89) | Ref |
|  | Female | 0.89 (0.85, 0.93) | 0.652 |
|  | Age |  |  |
|  | 0-4 years | 0.80 (0.63, 1.01) | Ref |
|  | 5-19 years | 0.87 (0.82, 0.92) | 0.975 |
|  | ≥20 years | 0.87 (0.83, 0.91) | 0.736 |
| Measles | Total | 0.12 (0.07, 0.20) |  |
|  | Sex |  |  |
|  | Male | 0.11 (0.06, 0.20) | Ref |
|  | Female | 0.11 (0.07, 0.20) | 0.986 |
|  | Age |  |  |
|  | 0-4 years | 0.18 (0.11, 0.30) | Ref |
|  | 5-19 years | 0.09 (0.05, 0.17) | 0.731 |
|  | ≥20 years | 0.06 (0.03, 0.14) | 0.291 |
| Scarlet fever | Total | 0.14 (0.11, 0.18) |  |
|  | Sex |  |  |
|  | Male | 0.14 (0.11, 0.17) | Ref |
|  | Female | 0.15 (0.12, 0.19) | 0.784 |
|  | Age |  |  |
|  | 0-4 years | 0.17 (0.14, 0.20) | Ref |
|  | 5-19 years | 0.12 (0.10, 0.16) | **< 0.001** |
|  | ≥20 years | 0.43 (0.35, 0.53) | **0.001** |
| Mumps | Total | 0.63 (0.55, 0.72) |  |
|  | Sex |  |  |
|  | Male | 0.63 (0.55, 0.72) | Ref |
|  | Female | 0.64 (0.56, 0.74) | 0.885 |
|  | Age |  |  |
|  | 0-4 years | 0.95 (0.87, 1.04) | Ref |
|  | 5-19 years | 0.55 (0.47, 0.66) | 0.328 |
|  | ≥20 years | 0.68 (0.61, 0.75) | **0.018** |
| Rubella | Total | 0.25 (0.12, 0.53) |  |
|  | Sex |  |  |
|  | Male | 0.21 (0.09, 0.47) | Ref |
|  | Female | 0.31 (0.15, 0.63) | 0.717 |
|  | Age |  |  |
|  | 0-4 years | 1.12 (0.83, 1.51) | Ref |
|  | 5-19 years | 0.21 (0.06, 0.69) | 0.985 |
|  | ≥20 years | 0.20 (0.12, 0.34) | **0.005** |
| Varicella | Total | 0.56 (0.52, 0.61) |  |
|  | Sex |  |  |
|  | Male | 0.57 (0.52, 0.61) | Ref |
|  | Female | 0.56 (0.52, 0.61) | 0.910 |
|  | Age |  |  |
|  | 0-4 years | 0.57 (0.51, 0.64) | Ref |
|  | 5-19 years | 0.56 (0.51, 0.61) | 0.838 |
|  | ≥20 years | 0.54 (0.51, 0.58) | 0.690 |
| Bacillary Dysentery | Total | 0.77 (0.72, 0.84) |  |
|  | Sex |  |  |
|  | Male | 0.78 (0.72, 0.85) | Ref |
|  | Female | 0.76 (0.71, 0.82) | 0.857 |
|  | Age |  |  |
|  | 0-4 years | 0.71 (0.64, 0.79) | Ref |
|  | 5-19 years | 0.87 (0.79, 0.96) | 0.494 |
|  | ≥20 years | 0.80 (0.74, 0.86) | 0.401 |
| Infectious diarrhea | Total | 0.68 (0.62, 0.75) |  |
|  | Sex |  |  |
|  | Male | 0.67 (0.61, 0.74) | Ref |
|  | Female | 0.69 (0.62, 0.76) | 0.875 |
|  | Age |  |  |
|  | 0-4 years | 0.59 (0.53, 0.66) | Ref |
|  | 5-19 years | 0.92 (0.83, 1.02) | 0.222 |
|  | ≥20 years | 0.76 (0.68, 0.85) | 0.110 |
| HFMD | Total | 0.25 (0.18, 0.34) |  |
|  | Sex |  |  |
|  | Male | 0.24 (0.18, 0.33) | Ref |
|  | Female | 0.26 (0.19, 0.35) | 0.866 |
|  | Age |  |  |
|  | 0-4 years | 0.25 (0.18, 0.34) | Ref |
|  | 5-19 years | 0.23 (0.16, 0.31) | 0.544 |
|  | ≥20 years | 0.30 (0.21, 0.43) | 0.698 |

^*^ Random effect meta regression model was used to test the significance of difference in relative risks. HFMD = Hand, foot, and mouth disease.

**
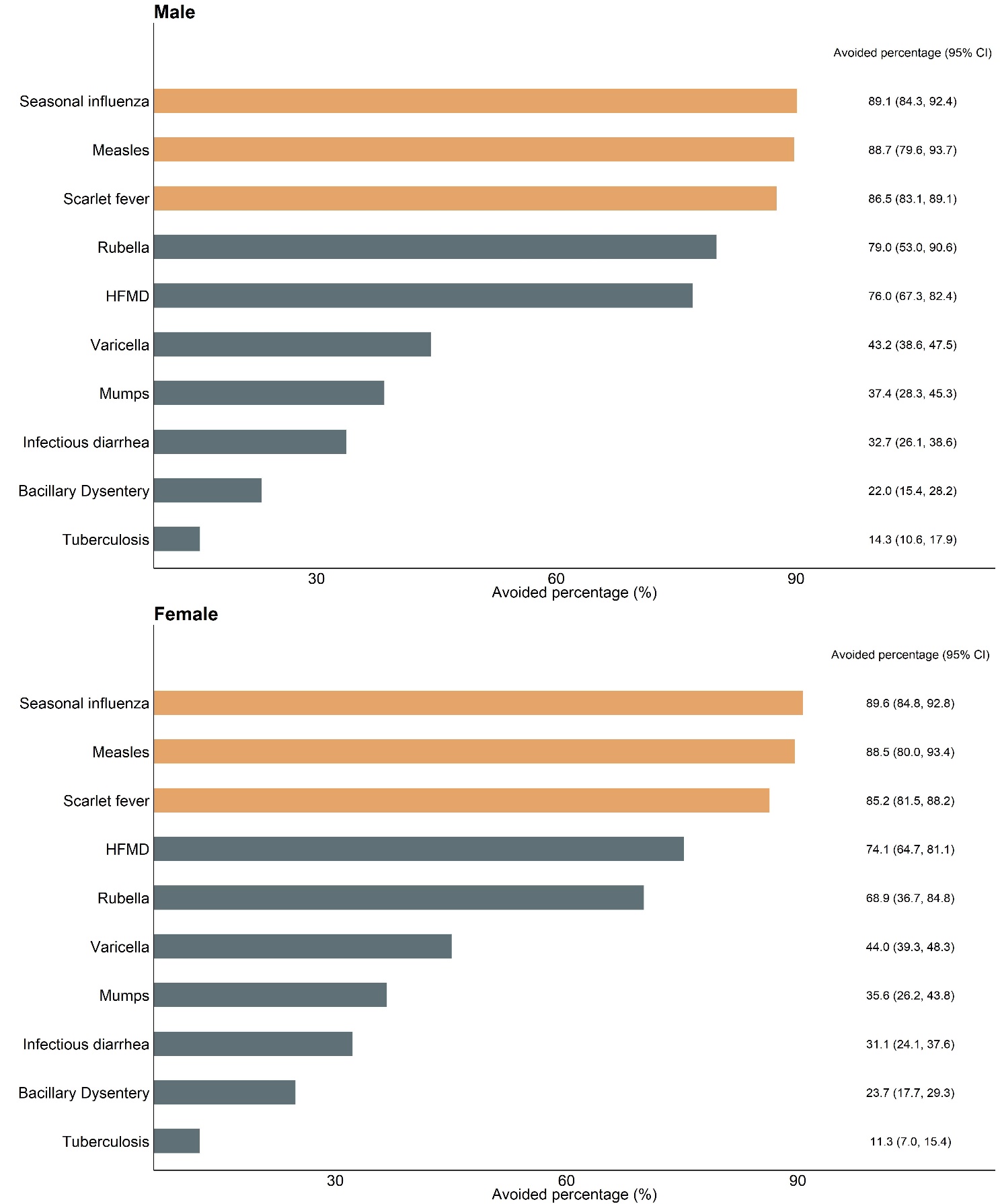
**

**Figure S3. Percentage of cases avoided associated with non-pharmaceutical interventions during COVID-19 pandemic in 2020, stratified by sex.** HFMD = Hand, foot, and mouth disease. Orange bars represent the top three diseases with the highest percentage of cases avoided.

**
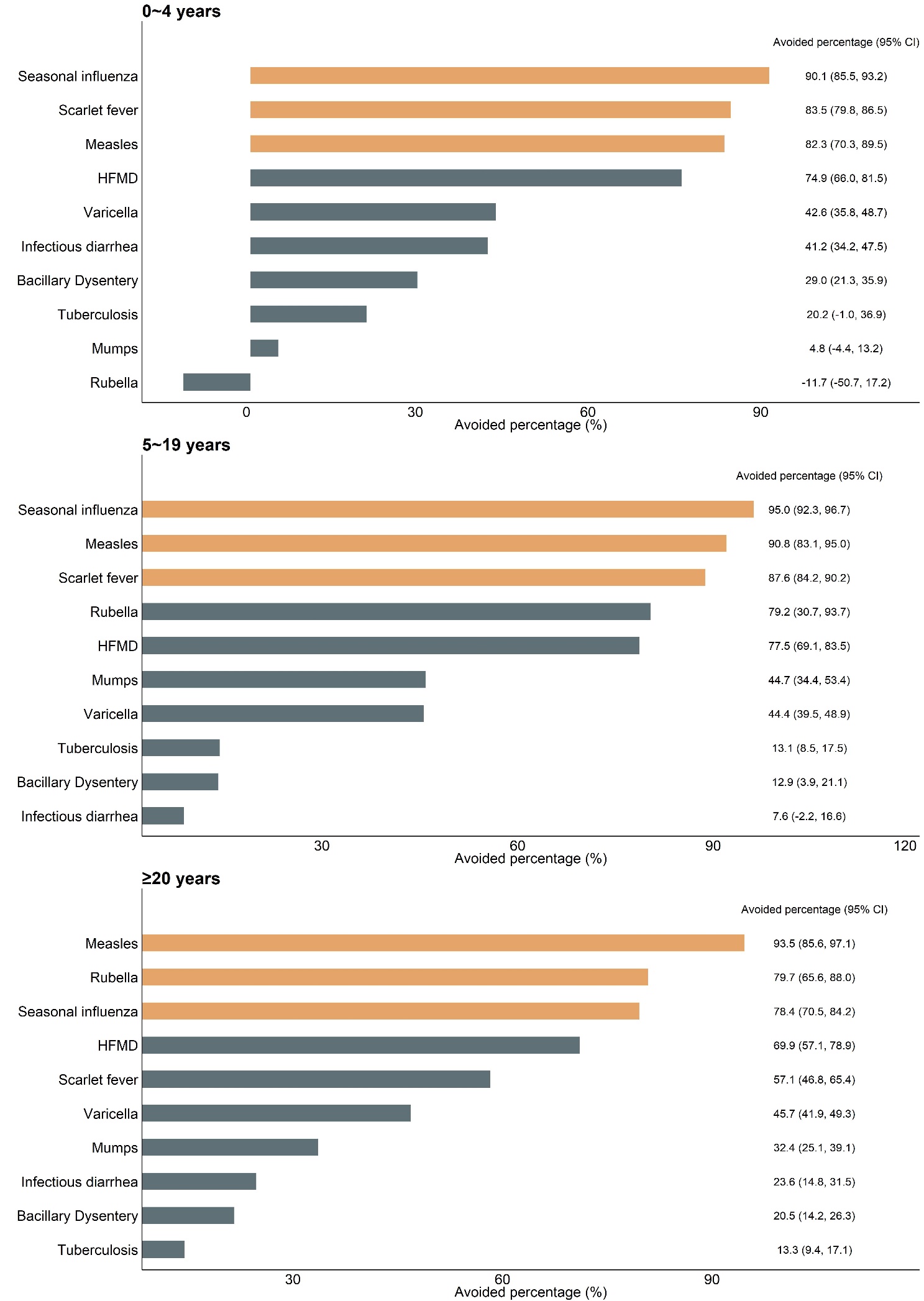
**

**Figure S4. Percentage of cases avoided associated with non-pharmaceutical interventions during COVID-19 pandemic in 2020, stratified by age.** HFMD = Hand, foot, and mouth disease. Orange bars represent the top three diseases with the highest percentage of cases avoided.

**Table S5. Pooled relative risks of infectious disease incidence associated with non-pharmaceutical interventions during the COVID-19 pandemic stratified by urbanization rate level.**

| **Diseases** | **Level of urbanization rate** | **Relative risk (95% *CI*)** | **P for difference ^*^** |
| --- | --- | --- | --- |
| Seasonal influenza | L | 0.19 (0.12, 0.32) | Ref |
|  | LM | 0.10 (0.05, 0.22) | 0.162 |
|  | HM | 0.15 (0.10, 0.24) | 0.474 |
|  | H | 0.04 (0.02, 0.09) | **0.001** |
| Tuberculosis | L | 0.83 (0.77, 0.91) | Ref |
|  | LM | 0.84 (0.76, 0.93) | 0.946 |
|  | HM | 0.87 (0.79, 0.95) | 0.578 |
|  | H | 0.94 (0.90, 0.98) | 0.068 |
| Measles | L | 0.08 (0.02, 0.29) | Ref |
|  | LM | 0.17 (0.07, 0.41) | 0.341 |
|  | HM | 0.11 (0.03, 0.37) | 0.685 |
|  | H | 0.08 (0.02, 0.27) | 0.997 |
| Scarlet fever | L | 0.20 (0.14, 0.28) | Ref |
|  | LM | 0.14 (0.08, 0.23) | 0.266 |
|  | HM | 0.12 (0.09, 0.16) | 0.052 |
|  | H | 0.11 (0.07, 0.20) | 0.117 |
| Mumps | L | 0.57 (0.44, 0.73) | Ref |
|  | LM | 0.53 (0.45, 0.62) | 0.652 |
|  | HM | 0.86 (0.67, 1.10) | **0.024** |
|  | H | 0.63 (0.46, 0.85) | 0.615 |
| Rubella | L | 0.20 (0.08, 0.51) | Ref |
|  | LM | 0.32 (0.03, 3.24) | 0.705 |
|  | HM | 0.27 (0.11, 0.65) | 0.637 |
|  | H | 0.16 (0.06, 0.41) | 0.741 |
| Varicella | L | 0.58 (0.48, 0.70) | Ref |
|  | LM | 0.58 (0.51, 0.65) | 0.993 |
|  | HM | 0.58 (0.51, 0.66) | 0.951 |
|  | H | 0.52 (0.43, 0.64) | 0.508 |
| Bacillary Dysentery | L | 0.78 (0.72, 0.85) | Ref |
|  | LM | 0.75 (0.68, 0.83) | 0.651 |
|  | HM | 0.74 (0.62, 0.88) | 0.605 |
|  | H | 0.83 (0.65, 1.07) | 0.642 |
| Infectious diarrhea | L | 0.71 (0.63, 0.81) | Ref |
|  | LM | 0.78 (0.65, 0.94) | 0.441 |
|  | HM | 0.61 (0.54, 0.69) | 0.122 |
|  | H | 0.63 (0.46, 0.85) | 0.472 |
| HFMD | L | 0.37 (0.20, 0.68) | Ref |
|  | LM | 0.26 (0.15, 0.46) | 0.420 |
|  | HM | 0.14 (0.07, 0.29) | 0.052 |
|  | H | 0.25 (0.14, 0.45) | 0.390 |

^*^ Random effect meta regression model was used to test the significance of difference in relative risks. L = Low; LM = Lower middle; HM = Higher middle; H = High; HFMD = Hand, foot, and mouth disease.

**Table S6. Pooled relative risks of infectious disease incidence associated with non-pharmaceutical interventions during the COVID-19 pandemic stratified by population density level.**

| **Diseases** | **Population density** | **Relative risk (95% *CI*)** | **P for difference ^*^** |
| --- | --- | --- | --- |
| Seasonal influenza | L | 0.21 (0.14, 0.32) | Ref |
|  | LM | 0.13 (0.06, 0.26) | 0.229 |
|  | HM | 0.09 (0.04, 0.18) | **0.045** |
|  | H | 0.05 (0.02, 0.12) | **0.005** |
| Tuberculosis | L | 0.76 (0.65, 0.88) | Ref |
|  | LM | 0.90 (0.86, 0.94) | 0.057 |
|  | HM | 0.91 (0.86, 0.96) | **0.046** |
|  | H | 0.89 (0.85, 0.94) | 0.064 |
| Measles | L | 0.12 (0.04, 0.37) | Ref |
|  | LM | 0.16 (0.04, 0.66) | 0.723 |
|  | HM | 0.13 (0.06, 0.28) | 0.940 |
|  | H | 0.05 (0.01, 0.25) | 0.389 |
| Scarlet fever | L | 0.15 (0.09, 0.24) | Ref |
|  | LM | 0.16 (0.10, 0.25) | 0.923 |
|  | HM | 0.17 (0.11, 0.24) | 0.747 |
|  | H | 0.09 (0.06, 0.14) | 0.138 |
| Mumps | L | 0.69 (0.51, 0.93) | Ref |
|  | LM | 0.67 (0.53, 0.85) | 0.884 |
|  | HM | 0.56 (0.40, 0.78) | 0.369 |
|  | H | 0.63 (0.53, 0.76) | 0.635 |
| Rubella | L | 0.81 (0.10, 6.73) | Ref |
|  | LM | 0.15 (0.04, 0.51) | 0.175 |
|  | HM | 0.14 (0.05, 0.39) | 0.142 |
|  | H | 0.21 (0.10, 0.45) | 0.240 |
| Varicella | L | 0.63 (0.51, 0.77) | Ref |
|  | LM | 0.60 (0.53, 0.68) | 0.751 |
|  | HM | 0.54 (0.50, 0.59) | 0.253 |
|  | H | 0.50 (0.42, 0.58) | 0.100 |
| Bacillary Dysentery | L | 0.73 (0.65, 0.82) | Ref |
|  | LM | 0.87 (0.73, 1.03) | 0.116 |
|  | HM | 0.79 (0.66, 0.94) | 0.471 |
|  | H | 0.71 (0.63, 0.80) | 0.769 |
| Infectious diarrhea | L | 0.67 (0.56, 0.79) | Ref |
|  | LM | 0.80 (0.66, 0.98) | 0.195 |
|  | HM | 0.68 (0.59, 0.79) | 0.900 |
|  | H | 0.58 (0.46, 0.71) | 0.305 |
| HFMD | L | 0.13 (0.05, 0.34) | Ref |
|  | LM | 0.22 (0.10, 0.48) | 0.389 |
|  | HM | 0.40 (0.27, 0.58) | **0.035** |
|  | H | 0.23 (0.14, 0.37) | 0.320 |

^*^ Random effect meta regression model was used to test the significance of difference in relative risks. L = Low; LM = Lower middle; HM = Higher middle; H = High; HFMD = Hand, foot, and mouth disease.

**Table S7. Pooled relative risks of infectious disease incidence associated with non-pharmaceutical interventions during the COVID-19 pandemic stratified by GDP per-capita level.**

| **Diseases** | **Level of GDP per capita** | **Relative risk (95% *CI*)** | **P for difference ^*^** |
| --- | --- | --- | --- |
| Seasonal influenza | L | 0.22 (0.16, 0.31) | Ref |
|  | LM | 0.12 (0.06, 0.24) | 0.120 |
|  | HM | 0.10 (0.05, 0.21) | 0.058 |
|  | H | 0.04 (0.02, 0.09) | **<0.001** |
| Tuberculosis | L | 0.80 (0.71, 0.89) | Ref |
|  | LM | 0.84 (0.75, 0.95) | 0.548 |
|  | HM | 0.90 (0.86, 0.94) | 0.105 |
|  | H | 0.93 (0.90, 0.98) | **0.032** |
| Measles | L | 0.06 (0.01, 0.36) | Ref |
|  | LM | 0.16 (0.07, 0.37) | 0.319 |
|  | HM | 0.11 (0.03, 0.35) | 0.577 |
|  | H | 0.08 (0.02, 0.27) | 0.790 |
| Scarlet fever | L | 0.15 (0.09, 0.25) | Ref |
|  | LM | 0.20 (0.13, 0.31) | 0.382 |
|  | HM | 0.12 (0.09, 0.17) | 0.591 |
|  | H | 0.10 (0.07, 0.16) | 0.306 |
| Mumps | L | 0.57 (0.48, 0.68) | Ref |
|  | LM | 0.62 (0.45, 0.86) | 0.638 |
|  | HM | 0.59 (0.41, 0.85) | 0.854 |
|  | H | 0.76 (0.65, 0.89) | **0.021** |
| Rubella | L | 0.39 (0.04, 3.73) | Ref |
|  | LM | 0.21 (0.08, 0.54) | 0.603 |
|  | HM | 0.13 (0.05, 0.35) | 0.368 |
|  | H | 0.26 (0.12, 0.57) | 0.733 |
| Varicella | L | 0.63 (0.56, 0.71) | Ref |
|  | LM | 0.57 (0.47, 0.71) | 0.488 |
|  | HM | 0.56 (0.50, 0.62) | 0.191 |
|  | H | 0.50 (0.42, 0.58) | **0.033** |
| Bacillary Dysentery | L | 0.76 (0.68, 0.85) | Ref |
|  | LM | 0.80 (0.66, 0.97) | 0.666 |
|  | HM | 0.72 (0.66, 0.78) | 0.481 |
|  | H | 0.82 (0.65, 1.03) | 0.587 |
| Infectious diarrhea | L | 0.80 (0.66, 0.97) | Ref |
|  | LM | 0.74 (0.63, 0.87) | 0.569 |
|  | HM | 0.64 (0.56, 0.72) | 0.075 |
|  | H | 0.55 (0.45, 0.68) | **0.016** |
| HFMD | L | 0.18 (0.08, 0.41) | Ref |
|  | LM | 0.28 (0.13, 0.61) | 0.445 |
|  | HM | 0.28 (0.15, 0.53) | 0.430 |
|  | H | 0.22 (0.16, 0.31) | 0.669 |

^*^ Random effect meta regression model was used to test the significance of difference in relative risks. L = Low; LM = Lower middle; HM = Higher middle; H = High; HFMD = Hand, foot, and mouth disease.

**Table S8. PLAD-specific relative risks of infectious disease incidence associated with non-pharmaceutical interventions during the COVID-19 pandemic stratified by GDP per-capita level.**

| PLAD | GDP level | Seasonal influenza | Tuberculosis | Measles | Scarlet fever | Mumps | Rubella | Varicella | Bacillary Dysentery | Infectious diarrhea | Hand, foot and mouth disease |
| --- | --- | --- | --- | --- | --- | --- | --- | --- | --- | --- | --- |
| Gansu | L | 0.40 (0.29, 0.54) | 0.86 (0.77, 0.95) | 0.08 (0.00, 4.48) | 0.15 (0.10, 0.21) | 0.52 (0.26, 1.05) | 0.08 (0.00, 2.89) | 0.74 (0.62, 0.89) | 0.78 (0.66, 0.91) | 0.77 (0.67, 0.88) | 0.21 (0.13, 0.34) |
| Guangxi | L | 0.19 (0.10, 0.36) | 0.87 (0.83, 0.91) | 0.10 (0.00, /) | 0.23 (0.16, 0.33) | 0.77 (0.50, 1.17) | 0.09 (0.00, 2.01) | 0.49 (0.41, 0.58) | 0.86 (0.70, 1.05) | 0.68 (0.59, 0.78) | 0.14 (0.06, 0.30) |
| Guizhou | L | 0.28 (0.17, 0.47) | 0.88 (0.83, 0.94) | 0.06 (0.00, 7.59) | 0.35 (0.26, 0.48) | 0.50 (0.34, 0.73) | 0.30 (0.00, 107.06) | 0.61 (0.51, 0.74) | 0.74 (0.57, 0.95) | 1.05 (0.87, 1.28) | 0.93 (0.59, 1.47) |
| Hebei | L | 0.13 (0.08, 0.22) | 0.84 (0.79, 0.89) | 0.04 (0.00, 87.14) | 0.08 (0.05, 0.13) | 0.49 (0.29, 0.82) | 0.15 (0.01, 3.72) | 0.51 (0.39, 0.66) | 0.68 (0.62, 0.75) | 0.87 (0.77, 0.98) | 0.13 (0.05, 0.33) |
| Heilongjiang | L | 0.15 (0.07, 0.32) | 0.64 (0.59, 0.70) | 2.34 (0.00, /) | 0.05 (0.02, 0.15) | 0.57 (0.31, 1.04) | 0.11 (0.00, 6.35) | 0.68 (0.51, 0.91) | 0.62 (0.54, 0.71) | 0.45 (0.35, 0.58) | 0.01 (0.00, 0.18) |
| Jilin | L | 0.06 (0.02, 0.21) | 0.80 (0.74, 0.87) | 0.02 (0.00, 9.62) | 0.05 (0.02, 0.15) | 0.78 (0.46, 1.34) | 0.05 (0.00, 384.21) | 0.60 (0.46, 0.79) | 1.16 (0.87, 1.55) | 0.67 (0.43, 1.04) | 0.02 (0.00, 0.43) |
| Qinghai | L | 0.27 (0.16, 0.48) | 0.60 (0.55, 0.67) | 0.03 (0.00, 1.24) | 0.34 (0.24, 0.48) | 0.52 (0.35, 0.77) | 51.09 (18.66, 139.92) | 0.73 (0.59, 0.89) | 0.78 (0.65, 0.94) | 1.07 (0.87, 1.33) | 0.29 (0.00, 87.22) |
| Shanxi | L | 0.28 (0.19, 0.42) | 0.93 (0.88, 0.99) | 0.07 (0.00, 5.08) | 0.11 (0.06, 0.20) | 0.50 (0.29, 0.86) | 0.12 (0.00, 17.67) | 0.71 (0.57, 0.90) | 0.75 (0.62, 0.90) | 0.96 (0.82, 1.12) | 0.23 (0.12, 0.44) |
| Hainan | LM | 0.04 (0.02, 0.10) | 1.00 (0.94, 1.06) | 0.13 (0.00, 13.65) | 0.14 (0.06, 0.37) | 0.43 (0.25, 0.77) | 0.11 (0.02, 0.70) | 0.47 (0.36, 0.62) | 0.72 (0.49, 1.05) | 0.74 (0.63, 0.88) | 0.10 (0.03, 0.31) |
| Henan | LM | 0.08 (0.04, 0.15) | 0.79 (0.76, 0.84) | 0.03 (0.00, 1.97) | 0.17 (0.11, 0.25) | 0.37 (0.26, 0.52) | 0.15 (0.01, 1.69) | 0.63 (0.50, 0.80) | 0.86 (0.74, 1.01) | 0.68 (0.57, 0.80) | 0.61 (0.36, 1.01) |
| Jiangxi | LM | 0.29 (0.20, 0.42) | 0.82 (0.78, 0.86) | 0.23 (0.07, 0.74) | 0.44 (0.27, 0.71) | 0.50 (0.35, 0.72) | 0.03 (0.00, 10.32) | 0.58 (0.48, 0.70) | 0.78 (0.65, 0.94) | 0.82 (0.68, 0.99) | 0.71 (0.48, 1.05) |
| Ningxia | LM | 0.18 (0.09, 0.38) | 0.83 (0.75, 0.92) | 0.19 (0.02, 1.57) | 0.12 (0.08, 0.18) | 1.36 (0.82, 2.24) | 0.00 (0.00, Inf) | 0.56 (0.45, 0.70) | 0.59 (0.46, 0.75) | 0.68 (0.57, 0.81) | 0.06 (0.03, 0.17) |
| Sichuan | LM | 0.04 (0.01, 0.30) | 0.97 (0.93, 1.01) | 0.11 (0.01, 1.74) | 0.30 (0.22, 0.41) | 0.65 (0.45, 0.94) | 0.13 (0.00, 4.49) | 0.81 (0.68, 0.95) | 1.33 (1.16, 1.52) | 1.20 (0.99, 1.46) | 0.47 (0.30, 0.74) |
| Tibet | LM | 0.18 (0.01, 3.62) | 0.79 (0.72, 0.85) | 0.01 (0.00, /) | 0.28 (0.14, 0.57) | 1.14 (0.55, 2.34) | 62.55 (0.09, 43345.93) | 0.51 (0.38, 0.67) | 0.81 (0.60, 1.09) | 0.74 (0.50, 1.08) | 0.52 (0.30, 0.89) |
| Xinjiang | LM | 0.05 (0.02, 0.15) | 0.49 (0.37, 0.66) | 0.01 (0.00, 38.44) | 0.06 (0.04, 0.11) | 0.42 (0.27, 0.65) | 0.44 (0.07, 2.58) | 0.34 (0.27, 0.43) | 0.59 (0.47, 0.74) | 0.56 (0.45, 0.70) | 0.03 (0.01, 0.14) |
| Yunnan | LM | 0.35 (0.23, 0.53) | 0.99 (0.95, 1.04) | 0.12 (0.02, 0.77) | 0.32 (0.24, 0.41) | 0.85 (0.55, 1.31) | 0.16 (0.01, 1.95) | 0.83 (0.71, 0.96) | 0.86 (0.73, 1.01) | 0.62 (0.54, 0.73) | 0.60 (0.43, 0.84) |
| Anhui | HM | 0.19 (0.12, 0.29) | 0.84 (0.79, 0.89) | 0.04 (0.00, 1.54) | 0.18 (0.12, 0.26) | 0.48 (0.29, 0.78) | 0.10 (0.01, 0.80) | 0.58 (0.48, 0.69) | 0.71 (0.57, 0.89) | 0.69 (0.60, 0.79) | 0.61 (0.39, 0.96) |
| Chongqing | HM | 0.11 (0.05, 0.28) | 0.94 (0.89, 0.99) | 0.02 (0.00, 5.25) | 0.21 (0.14, 0.31) | 0.68 (0.51, 0.91) | 0.05 (0.00, 1.36) | 0.46 (0.39, 0.55) | 0.61 (0.50, 0.74) | 0.66 (0.52, 0.83) | 0.33 (0.22, 0.49) |
| Hubei | HM | 0.02 (0.01, 0.06) | 0.89 (0.83, 0.95) | 0.05 (0.00, 0.80) | 0.07 (0.04, 0.12) | 0.43 (0.27, 0.67) | 0.12 (0.00, 3.65) | 0.45 (0.35, 0.58) | 0.63 (0.53, 0.75) | 0.45 (0.36, 0.55) | 0.26 (0.17, 0.42) |
| Hunan | HM | 0.02 (0.01, 0.07) | 0.99 (0.94, 1.04) | 0.06 (0.00, 1.57) | 0.20 (0.14, 0.27) | 0.26 (0.19, 0.37) | 0.06 (0.00, 1.85) | 0.52 (0.44, 0.62) | 0.80 (0.64, 1.00) | 0.71 (0.56, 0.90) | 0.80 (0.54, 1.17) |
| Inner Mongolia | HM | 0.16 (0.09, 0.27) | 0.87 (0.80, 0.96) | 0.15 (0.01, 3.87) | 0.10 (0.06, 0.17) | 0.69 (0.34, 1.39) | 0.22 (0.02, 2.36) | 0.77 (0.60, 0.98) | 0.87 (0.70, 1.07) | 0.61 (0.52, 0.72) | 0.06 (0.03, 0.14) |
| Liaoning | HM | 0.32 (0.20, 0.50) | 0.86 (0.79, 0.93) | 0.15 (0.00, /) | 0.07 (0.03, 0.15) | 1.64 (0.87, 3.09) | 2.33 (0.04, 139.49) | 0.57 (0.45, 0.71) | 0.75 (0.66, 0.86) | 0.51 (0.42, 0.62) | 0.02 (0.00, 0.17) |
| Shaanxi | HM | 0.02 (0.00, 0.13) | 0.87 (0.82, 0.91) | 0.46 (0.05, 4.31) | 0.12 (0.07, 0.19) | 0.75 (0.43, 1.29) | 0.05 (0.00, 27.84) | 0.57 (0.46, 0.70) | 0.78 (0.64, 0.96) | 0.78 (0.61, 1.01) | 0.44 (0.27, 0.70) |
| Shandong | HM | 0.25 (0.16, 0.37) | 0.92 (0.88, 0.96) | 0.06 (0.00, 2.22) | 0.09 (0.06, 0.13) | 0.65 (0.37, 1.14) | 0.11 (0.01, 0.85) | 0.62 (0.50, 0.77) | 0.63 (0.50, 0.79) | 0.75 (0.67, 0.84) | 0.27 (0.16, 0.47) |
| Beijing | H | 0.01 (0.00, 0.03) | 0.93 (0.86, 1.00) | 0.05 (0.00, 6.78) | 0.05 (0.01, 0.15) | 0.64 (0.50, 0.82) | 0.30 (0.06, 1.58) | 0.59 (0.49, 0.73) | 0.62 (0.49, 0.80) | 0.37 (0.28, 0.49) | 0.09 (0.03, 0.29) |
| Fujian | H | 0.17 (0.11, 0.27) | 1.01 (0.95, 1.07) | 0.10 (0.01, 0.94) | 0.17 (0.12, 0.25) | 1.08 (0.60, 1.93) | 0.08 (0.00, 1.63) | 0.58 (0.47, 0.71) | 1.10 (0.90, 1.34) | 0.63 (0.47, 0.85) | 0.31 (0.19, 0.51) |
| Guangdong | H | 0.04 (0.01, 0.10) | 0.84 (0.79, 0.90) | 0.08 (0.00, 2.68) | 0.10 (0.06, 0.17) | 0.72 (0.49, 1.07) | 0.11 (0.02, 0.81) | 0.35 (0.28, 0.43) | 0.59 (0.52, 0.68) | 0.71 (0.56, 0.90) | 0.16 (0.08, 0.30) |
| Jiangsu | H | 0.06 (0.02, 0.20) | 0.96 (0.91, 1.00) | 0.02 (0.00, 9.65) | 0.16 (0.11, 0.23) | 0.71 (0.42, 1.22) | 0.35 (0.08, 1.44) | 0.45 (0.36, 0.56) | 0.80 (0.67, 0.95) | 0.55 (0.41, 0.72) | 0.33 (0.19, 0.55) |
| Shanghai | H | 0.05 (0.01, 0.20) | 0.92 (0.86, 0.99) | 0.04 (0.00, 2.28) | 0.08 (0.04, 0.16) | 0.67 (0.55, 0.81) | 0.22 (0.01, 3.53) | 0.44 (0.37, 0.52) | 1.00 (0.41, 2.46) | 0.37 (0.27, 0.51) | 0.14 (0.06, 0.32) |
| Tianjin | H | 0.02 (0.00, 0.30) | 0.91 (0.83, 0.99) | 0.07 (0.00, 17.81) | 0.04 (0.02, 0.07) | 0.76 (0.59, 0.98) | 0.13 (0.00, 6.31) | 0.46 (0.37, 0.59) | 0.72 (0.62, 0.83) | 0.67 (0.57, 0.79) | 0.14 (0.05, 0.36) |
| Zhejiang | H | 0.01 (0.00, 0.07) | 0.97 (0.92, 1.01) | 0.10 (0.01, 0.82) | 0.17 (0.10, 0.28) | 1.05 (0.80, 1.38) | 0.77 (0.08, 7.30) | 0.64 (0.55, 0.75) | 1.25 (1.00, 1.55) | 0.65 (0.49, 0.86) | 0.32 (0.20, 0.53) |

L = Low; LM = Lower middle; HM = Higher middle; H = High; HFMD = Hand, foot, and mouth disease.

**Table S9. Pooled relative risks of infectious disease incidence associated with non-pharmaceutical interventions during the COVID-19 pandemic stratified by non-pharmaceutical interventions level.**

| **Diseases** | **Level of non-pharmaceutical interventions** | **Relative risk (95% *CI*)** | **P for difference ^*^** |
| --- | --- | --- | --- |
| Seasonal influenza | L | 0.12 (0.08, 0.17) | Ref |
|  | M | 0.09 (0.04, 0.17) | 0.427 |
|  | H | 0.23 (0.13, 0.41) | **0.046** |
| Tuberculosis | L | 0.90 (0.86, 0.94) | Ref |
|  | M | 0.84 (0.79, 0.90) | 0.238 |
|  | H | 0.77 (0.71, 0.82) | **0.007** |
| Measles | L | 0.14 (0.08, 0.24) | Ref |
|  | M | 0.16 (0.01, 1.88) | 0.925 |
|  | H | 0.00 (0.00, 12878.57) | 0.406 |
| Scarlet fever | L | 0.15 (0.12, 0.18) | Ref |
|  | M | 0.07 (0.04, 0.11) | **0.007** |
|  | H | 0.18 (0.11, 0.29) | 0.461 |
| Mumps | L | 0.67 (0.57, 0.77) | Ref |
|  | M | 0.41 (0.31, 0.53) | **0.003** |
|  | H | 0.64 (0.48, 0.86) | 0.810 |
| Rubella | L | 0.27 (0.12, 0.61) | Ref |
|  | M | 0.33 (0.03, 3.26) | 0.880 |
|  | H | 0.77 (0.32, 1.82) | 0.088 |
| Varicella | L | 0.59 (0.54, 0.64) | Ref |
|  | M | 0.25 (0.19, 0.32) | **<0.001** |
|  | H | 0.47 (0.39, 0.56) | **0.038** |
| Bacillary Dysentery | L | 0.81 (0.75, 0.87) | Ref |
|  | M | 0.73 (0.62, 0.87) | 0.385 |
|  | H | 0.51 (0.43, 0.59) | **<0.001** |
| Infectious diarrhea | L | 0.77 (0.71, 0.84) | Ref |
|  | M | 0.44 (0.34, 0.57) | **<0.001** |
|  | H | 0.29 (0.24, 0.36) | **<0.001** |
| HFMD | L | 0.27 (0.20, 0.36) | Ref |
|  | M | 0.04 (0.02, 0.11) | **<0.001** |
|  | H | 0.13 (0.07, 0.22) | **0.019** |

^*^ Random effect meta regression model was used to test the significance of difference in relative risks. L = Low; M = Middle; H = High; HFMD = Hand, foot, and mouth disease.

**Table S10. Pooled relative risks of infectious disease incidence associated with non-pharmaceutical interventions during the COVID-19 pandemic, using alternative degrees of freedom for mean temperature.**

| **Diseases** | **Degrees of freedom for mean temperature** | **Relative risk (95% *CI*)** | **P for difference ^*^** |
| --- | --- | --- | --- |
| Seasonal influenza | 3 ^#^ | 0.11 (0.07, 0.15) | Ref |
|  | 4 | 0.11 (0.07, 0.16) | 0.999 |
|  | 5 | 0.11 (0.07, 0.15) | 0.979 |
|  | 6 | 0.11 (0.07, 0.16) | 0.999 |
| Tuberculosis | 3 ^#^ | 0.87 (0.83, 0.91) | Ref |
|  | 4 | 0.87 (0.83, 0.91) | 0.992 |
|  | 5 | 0.87 (0.83, 0.91) | 0.994 |
|  | 6 | 0.87 (0.83, 0.91) | 0.979 |
| Measles | 3 ^#^ | 0.12 (0.07, 0.20) | Ref |
|  | 4 | 0.12 (0.07, 0.20) | 0.995 |
|  | 5 | 0.11 (0.06, 0.19) | 0.926 |
|  | 6 | 0.11 (0.06, 0.19) | 0.941 |
| Scarlet fever | 3 ^#^ | 0.14 (0.11, 0.18) | Ref |
|  | 4 | 0.14 (0.11, 0.18) | 0.995 |
|  | 5 | 0.14 (0.11, 0.18) | 0.995 |
|  | 6 | 0.14 (0.11, 0.18) | 0.999 |
| Mumps | 3 ^#^ | 0.63 (0.55, 0.72) | Ref |
|  | 4 | 0.63 (0.55, 0.72) | 0.989 |
|  | 5 | 0.64 (0.55, 0.73) | 0.986 |
|  | 6 | 0.63 (0.55, 0.73) | 0.996 |
| Rubella | 3 ^#^ | 0.25 (0.12, 0.53) | Ref |
|  | 4 | 0.24 (0.11, 0.51) | 0.973 |
|  | 5 | 0.25 (0.12, 0.53) | 0.997 |
|  | 6 | 0.25 (0.12, 0.52) | 0.999 |
| Varicella | 3 ^#^ | 0.56 (0.52, 0.61) | Ref |
|  | 4 | 0.56 (0.52, 0.61) | 0.966 |
|  | 5 | 0.56 (0.52, 0.61) | 0.969 |
|  | 6 | 0.56 (0.52, 0.61) | 0.968 |
| Bacillary Dysentery | 3 ^#^ | 0.77 (0.72, 0.84) | Ref |
|  | 4 | 0.77 (0.71, 0.83) | 0.978 |
|  | 5 | 0.77 (0.72, 0.84) | 0.993 |
|  | 6 | 0.77 (0.72, 0.84) | 0.990 |
| Infectious diarrhea | 3 ^#^ | 0.68 (0.62, 0.75) | Ref |
|  | 4 | 0.68 (0.62, 0.75) | 0.990 |
|  | 5 | 0.69 (0.63, 0.76) | 0.925 |
|  | 6 | 0.69 (0.63, 0.76) | 0.921 |
| HFMD | 3 ^#^ | 0.25 (0.18, 0.34) | Ref |
|  | 4 | 0.25 (0.18, 0.34) | 0.988 |
|  | 5 | 0.25 (0.18, 0.34) | 1.000 |
|  | 6 | 0.25 (0.18, 0.34) | 0.997 |

^#^ Degrees of freedom used in the main model. ^*^ Random effect meta regression model was used to test the significance of difference in relative risks. L = Low; M = Middle; H = High; HFMD = Hand, foot, and mouth disease.

**Table S11. Pooled relative risks of infectious disease incidence associated with non-pharmaceutical interventions during the COVID-19 pandemic, using alternative degrees of freedom for mean relative humidity.**

| **Diseases** | **Degrees of freedom for mean relative humidity** | **Relative risk (95% *CI*)** | **P for difference ^*^** |
| --- | --- | --- | --- |
| Seasonal influenza | 3 ^#^ | 0.11 (0.07, 0.15) | Ref |
|  | 4 | 0.10 (0.07, 0.15) | 0.962 |
|  | 5 | 0.11 (0.08, 0.15) | 0.992 |
|  | 6 | 0.11 (0.08, 0.16) | 0.902 |
| Tuberculosis | 3 ^#^ | 0.87 (0.83, 0.91) | Ref |
|  | 4 | 0.87 (0.83, 0.91) | 0.994 |
|  | 5 | 0.87 (0.83, 0.91) | 0.997 |
|  | 6 | 0.87 (0.83, 0.91) | 0.997 |
| Measles | 3 ^#^ | 0.12 (0.07, 0.20) | Ref |
|  | 4 | 0.11 (0.07, 0.20) | 0.989 |
|  | 5 | 0.11 (0.07, 0.20) | 0.982 |
|  | 6 | 0.11 (0.06, 0.19) | 0.941 |
| Scarlet fever | 3 ^#^ | 0.14 (0.11, 0.18) | Ref |
|  | 4 | 0.14 (0.11, 0.18) | 0.991 |
|  | 5 | 0.14 (0.11, 0.18) | 0.994 |
|  | 6 | 0.14 (0.11, 0.17) | 0.981 |
| Mumps | 3 ^#^ | 0.63 (0.55, 0.72) | Ref |
|  | 4 | 0.63 (0.55, 0.73) | 0.998 |
|  | 5 | 0.63 (0.55, 0.73) | 0.999 |
|  | 6 | 0.63 (0.55, 0.72) | 0.963 |
| Rubella | 3 ^#^ | 0.25 (0.12, 0.53) | Ref |
|  | 4 | 0.23 (0.11, 0.50) | 0.950 |
|  | 5 | 0.23 (0.11, 0.48) | 0.927 |
|  | 6 | 0.22 (0.11, 0.47) | 0.916 |
| Varicella | 3 ^#^ | 0.56 (0.52, 0.61) | Ref |
|  | 4 | 0.56 (0.52, 0.61) | 0.942 |
|  | 5 | 0.56 (0.52, 0.61) | 0.950 |
|  | 6 | 0.56 (0.52, 0.61) | 0.957 |
| Bacillary Dysentery | 3 ^#^ | 0.77 (0.72, 0.84) | Ref |
|  | 4 | 0.77 (0.72, 0.84) | 0.997 |
|  | 5 | 0.77 (0.72, 0.84) | 0.997 |
|  | 6 | 0.77 (0.72, 0.84) | 0.997 |
| Infectious diarrhea | 3 ^#^ | 0.68 (0.62, 0.75) | Ref |
|  | 4 | 0.68 (0.62, 0.75) | 0.979 |
|  | 5 | 0.68 (0.62, 0.75) | 0.972 |
|  | 6 | 0.68 (0.62, 0.75) | 0.972 |
| HFMD | 3 ^#^ | 0.25 (0.18, 0.34) | Ref |
|  | 4 | 0.25 (0.18, 0.34) | 0.992 |
|  | 5 | 0.25 (0.18, 0.34) | 0.997 |
|  | 6 | 0.25 (0.18, 0.34) | 0.997 |

^#^ Degrees of freedom used in the main model. ^*^ Random effect meta regression model was used to test the significance of difference in relative risks. L = Low; M = Middle; H = High; HFMD = Hand, foot, and mouth disease.

**Table S12. Pooled relative risks of infectious disease incidence associated with non-pharmaceutical interventions during the COVID-19 pandemic, using alternative degrees of freedom for mean precipitation.**

| **Diseases** | **Degrees of freedom for mean precipitation** | **Relative risk (95% *CI*)** | **P for difference ^*^** |
| --- | --- | --- | --- |
| Seasonal influenza | 3 ^#^ | 0.11 (0.07, 0.15) | Ref |
|  | 4 | 0.11 (0.08, 0.16) | 0.955 |
|  | 5 | 0.11 (0.08, 0.16) | 0.920 |
|  | 6 | 0.11 (0.08, 0.16) | 0.890 |
| Tuberculosis | 3 ^#^ | 0.87 (0.83, 0.91) | Ref |
|  | 4 | 0.87 (0.83, 0.91) | 0.995 |
|  | 5 | 0.87 (0.83, 0.91) | 0.997 |
|  | 6 | 0.87 (0.83, 0.91) | 0.999 |
| Measles | 3 ^#^ | 0.12 (0.07, 0.20) | Ref |
|  | 4 | 0.11 (0.06, 0.19) | 0.956 |
|  | 5 | 0.12 (0.07, 0.20) | 1.000 |
|  | 6 | 0.11 (0.06, 0.19) | 0.939 |
| Scarlet fever | 3 ^#^ | 0.14 (0.11, 0.18) | Ref |
|  | 4 | 0.14 (0.11, 0.18) | 0.983 |
|  | 5 | 0.14 (0.11, 0.18) | 0.989 |
|  | 6 | 0.14 (0.11, 0.18) | 0.986 |
| Mumps | 3 ^#^ | 0.63 (0.55, 0.72) | Ref |
|  | 4 | 0.63 (0.55, 0.72) | 0.989 |
|  | 5 | 0.63 (0.55, 0.72) | 0.991 |
|  | 6 | 0.63 (0.55, 0.72) | 0.993 |
| Rubella | 3 ^#^ | 0.25 (0.12, 0.53) | Ref |
|  | 4 | 0.26 (0.12, 0.54) | 0.983 |
|  | 5 | 0.27 (0.12, 0.58) | 0.952 |
|  | 6 | 0.27 (0.12, 0.57) | 0.953 |
| Varicella | 3 ^#^ | 0.56 (0.52, 0.61) | Ref |
|  | 4 | 0.56 (0.52, 0.61) | 0.999 |
|  | 5 | 0.56 (0.52, 0.61) | 0.974 |
|  | 6 | 0.56 (0.52, 0.61) | 0.976 |
| Bacillary Dysentery | 3 ^#^ | 0.77 (0.72, 0.84) | Ref |
|  | 4 | 0.77 (0.72, 0.84) | 0.991 |
|  | 5 | 0.78 (0.72, 0.84) | 0.987 |
|  | 6 | 0.78 (0.72, 0.84) | 0.986 |
| Infectious diarrhea | 3 ^#^ | 0.68 (0.62, 0.75) | Ref |
|  | 4 | 0.68 (0.62, 0.75) | 0.998 |
|  | 5 | 0.68 (0.62, 0.75) | 0.986 |
|  | 6 | 0.68 (0.62, 0.75) | 0.985 |
| HFMD | 3 ^#^ | 0.25 (0.18, 0.34) | Ref |
|  | 4 | 0.25 (0.18, 0.34) | 0.995 |
|  | 5 | 0.25 (0.18, 0.34) | 0.986 |
|  | 6 | 0.24 (0.18, 0.34) | 0.979 |

^#^ Degrees of freedom used in the main model. ^*^ Random effect meta regression model was used to test the significance of difference in relative risks. L = Low; M = Middle; H = High; HFMD = Hand, foot, and mouth disease.

**Table S13. Pooled relative risks of infectious disease incidence associated with non-pharmaceutical interventions during the COVID-19 pandemic, using alternative lag days for meteorological factors.**

| **Diseases** | **Lag days for all meteorological factors** | **Relative risk (95% *CI*)** | **P for difference** |
| --- | --- | --- | --- |
| Seasonal influenza | Lag 0 ^#^ | 0.11 (0.07, 0.15) | Ref |
|  | Lag 1 | 0.11 (0.08, 0.16) | 0.955 |
|  | Lag 2 | 0.12 (0.09, 0.17) | 0.798 |
| Tuberculosis | Lag 0 ^#^ | 0.87 (0.83, 0.91) | Ref |
|  | Lag 1 | 0.87 (0.83, 0.91) | 0.954 |
|  | Lag 2 | 0.87 (0.83, 0.91) | 0.959 |
| Measles | Lag 0 ^#^ | 0.12 (0.07, 0.20) | Ref |
|  | Lag 1 | 0.12 (0.07, 0.21) | 0.946 |
|  | Lag 2 | 0.13 (0.08, 0.22) | 0.859 |
| Scarlet fever | Lag 0 ^#^ | 0.14 (0.11, 0.18) | Ref |
|  | Lag 1 | 0.14 (0.11, 0.17) | 0.966 |
|  | Lag 2 | 0.14 (0.11, 0.17) | 0.978 |
| Mumps | Lag 0 ^#^ | 0.63 (0.55, 0.72) | Ref |
|  | Lag 1 | 0.64 (0.55, 0.73) | 0.988 |
|  | Lag 2 | 0.62 (0.54, 0.71) | 0.931 |
| Rubella | Lag 0 ^#^ | 0.25 (0.12, 0.53) | Ref |
|  | Lag 1 | 0.21 (0.13, 0.34) | 0.849 |
|  | Lag 2 | 0.24 (0.12, 0.47) | 0.955 |
| Varicella | Lag 0 ^#^ | 0.56 (0.52, 0.61) | Ref |
|  | Lag 1 | 0.56 (0.51, 0.60) | 0.889 |
|  | Lag 2 | 0.55 (0.51, 0.60) | 0.834 |
| Bacillary Dysentery | Lag 0 ^#^ | 0.77 (0.72, 0.84) | Ref |
|  | Lag 1 | 0.78 (0.72, 0.84) | 0.977 |
|  | Lag 2 | 0.78 (0.72, 0.84) | 0.985 |
| Infectious diarrhea | Lag 0 ^#^ | 0.68 (0.62, 0.75) | Ref |
|  | Lag 1 | 0.68 (0.62, 0.74) | 0.963 |
|  | Lag 2 | 0.67 (0.61, 0.73) | 0.895 |
| HFMD | Lag 0 ^#^ | 0.25 (0.18, 0.34) | Ref |
|  | Lag 1 | 0.25 (0.18, 0.34) | 0.976 |
|  | Lag 2 | 0.25 (0.18, 0.34) | 0.993 |

^#^ Degrees of freedom used in the main model. ^*^ Random effect meta regression model was used to test the significance of difference in relative risks. L = Low; M = Middle; H = High; HFMD = Hand, foot, and mouth disease.

**Table S14. Pooled relative risks of infectious disease incidence associated with non-pharmaceutical interventions during the COVID-19 pandemic, using different study periods.**

| **Diseases** | **Study period** | **Relative risk (95% *CI*)** | **P for difference ^*^** |
| --- | --- | --- | --- |
| Seasonal influenza | 2010-2020 ^#^ | 0.11 (0.07, 0.15) | Ref |
|  | 2017-2020 | 0.05 (0.03, 0.08) | 0.226 |
| Measles | 2010-2020 ^#^ | 0.12 (0.07, 0.20) | Ref |
|  | 2017-2020 | 0.06 (0.00, 2.35) | 0.849 |
| HFMD | 2010-2020 ^#^ | 0.25 (0.18, 0.34) | Ref |
|  | 2017-2020 | 0.18 (0.11, 0.28) | 0.545 |
| Scarlet fever | 2010-2020 ^#^ | 0.14 (0.11, 0.18) | Ref |
|  | 2017-2020 | 0.15 (0.12, 0.19) | 0.871 |
| Varicella | 2010-2020 ^#^ | 0.56 (0.52, 0.61) | Ref |
|  | 2017-2020 | 0.50 (0.46, 0.54) | 0.307 |
| Bacillary Dysentery | 2010-2020 ^#^ | 0.77 (0.72, 0.84) | Ref |
|  | 2017-2020 | 0.79 (0.73, 0.86) | 0.831 |
| Mumps | 2010-2020 ^#^ | 0.63 (0.55, 0.72) | Ref |
|  | 2017-2020 | 0.41 (0.35, 0.48) | **0.046** |
| Tuberculosis | 2010-2020 ^#^ | 0.87 (0.83, 0.91) | Ref |
|  | 2017-2020 | 0.95 (0.92, 0.97) | 0.193 |
| Infectious diarrhea | 2010-2020 ^#^ | 0.68 (0.62, 0.75) | Ref |
|  | 2017-2020 | 0.75 (0.68, 0.82) | 0.495 |
| Rubella | 2010-2020 ^#^ | 0.25 (0.12, 0.53) | Ref |
|  | 2017-2020 | 0.01 (0.01, 0.02) | **<0.001** |

^#^ Degrees of freedom used in the main model. ^*^ Random effect meta regression model was used to test the significance of difference in relative risks.


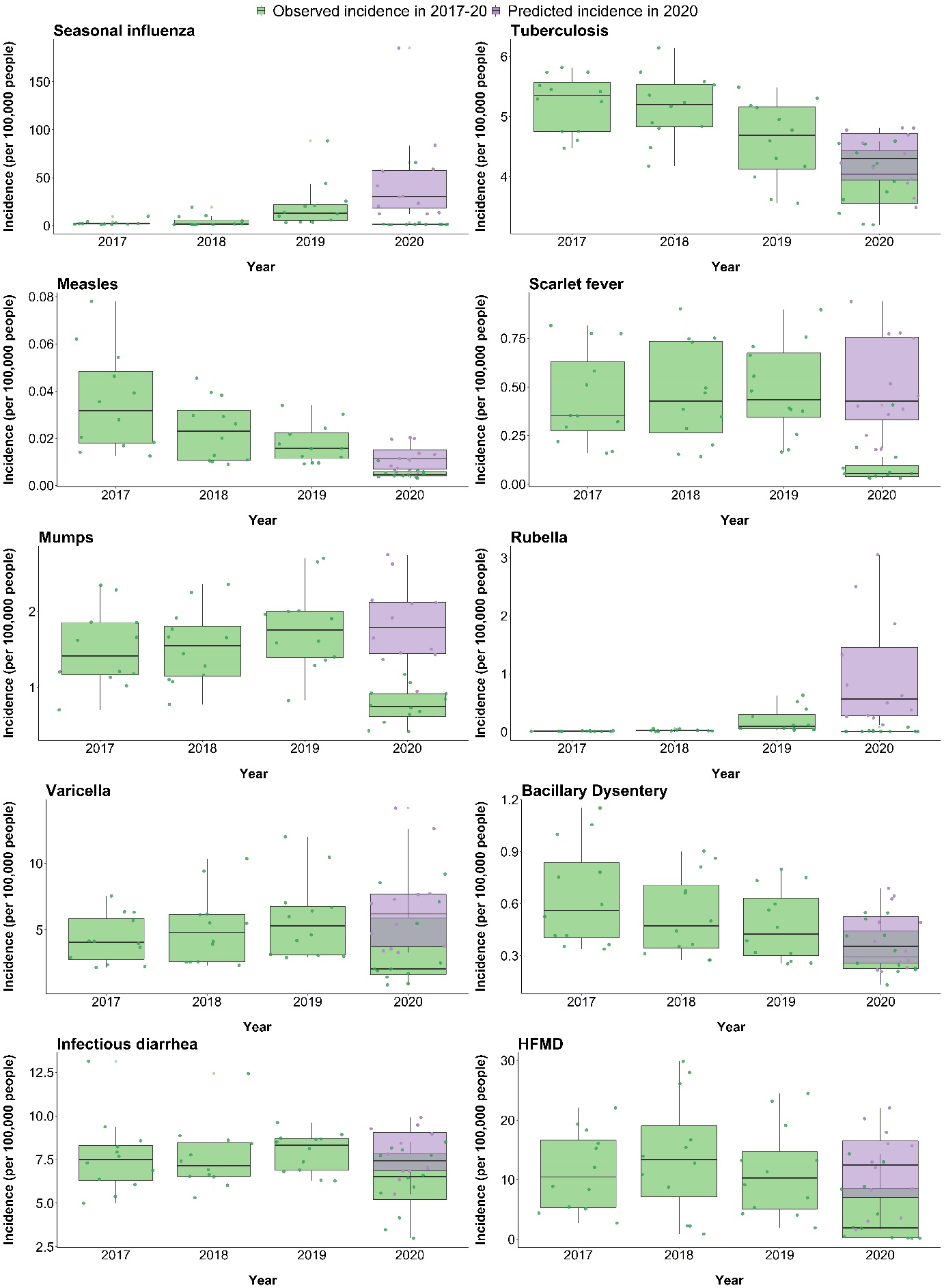


**Figure S5. Annual incidence rates from 2010 to 2020 (green-colored boxes) and predicted incidence rates without non-pharmaceutical interventions in 2020 using controlled interrupted time series model (purple-colored boxes) for ten infectious diseases.** HFMD = Hand, foot, and mouth disease.

**Table S15. Pooled relative risks of infectious disease incidence associated with non-pharmaceutical interventions during the COVID-19 pandemic, stratified by age groups.**

| **Diseases** | **Subgroup** | **Relative risk (95% *CI*)** | **P for difference ^*^** |
| --- | --- | --- | --- |
| Seasonal influenza | All ages | 0.11 (0.07, 0.15) |  |
|  | 0-4 years | 0.10 (0.07, 0.14) | Ref |
|  | 5-19 years | 0.05 (0.03, 0.08) | 0.234 |
|  | 20-54 years | 0.18 (0.13, 0.25) | 0.242 |
|  | ≥55 years | 0.30 (0.23, 0.40) | **0.016** |
| Tuberculosis | All ages | 0.87 (0.83, 0.91) |  |
|  | 0-4 years | 0.80 (0.63, 1.01) | Ref |
|  | 5-19 years | 0.87 (0.82, 0.92) | 0.730 |
|  | 20-54 years | 0.85 (0.82, 0.89) | 0.786 |
|  | ≥55 years | 0.87 (0.83, 0.92) | 0.710 |
| Mumps | All ages | 0.63 (0.55, 0.72) |  |
|  | 0-4 years | 0.95 (0.87, 1.04) | Ref |
|  | 5-19 years | 0.55 (0.47, 0.66) | **0.006** |
|  | 20-54 years | 0.64 (0.57, 0.72) | **0.010** |
|  | ≥55 years | 0.77 (0.72, 0.82) | **0.076** |
| Varicella | All ages | 0.56 (0.52, 0.61) |  |
|  | 0-4 years | 0.57 (0.51, 0.64) | Ref |
|  | 5-19 years | 0.56 (0.51, 0.61) | 0.832 |
|  | 20-54 years | 0.54 (0.50, 0.58) | 0.654 |
|  | ≥55 years | 0.83 (0.75, 0.93) | **0.019** |
| Bacillary Dysentery | All ages | 0.77 (0.72, 0.84) |  |
|  | 0-4 years | 0.71 (0.64, 0.79) | Ref |
|  | 5-19 years | 0.87 (0.79, 0.96) | 0.171 |
|  | 20-54 years | 0.78 (0.72, 0.84) | 0.495 |
|  | ≥55 years | 0.80 (0.74, 0.87) | 0.388 |
| Infectious diarrhea | All ages | 0.68 (0.62, 0.75) |  |
|  | 0-4 years | 0.59 (0.53, 0.66) | Ref |
|  | 5-19 years | 0.92 (0.83, 1.02) | **0.004** |
|  | 20-54 years | 0.76 (0.68, 0.85) | 0.121 |
|  | ≥55 years | 0.76 (0.68, 0.84) | 0.128 |

^*^ Random effect meta regression model was used to test the significance of difference in relative risks.

**Reference**

1. Chen J, Jia H, Cai Z, Zhou Y, Ma S, Chen Y *et al*: **Economic burden of measles and its influencing factors in Fujian, China**. *Hum Vaccin Immunother* 2021:1-5.

2. Lina Zhao, Jiangrong Li, Yujuan Chen, Ying Li, Wen Yu, Tang T: **Economic burden of mumps in Yunnan province, 2015-2017**. *Chinese Journal of Vaccines and Immunization* 2021, **27**(2):177-179,195. (in Chinese)

3. Shicheng Guo, Xiaofeng Liang, Zuo S: **Economical Burden on Measles, Rubbella, and Mumps' Cases in China in 2004**. *Chinses Journal of Vaccines and Immunization* 2008, **14**(1):32-36. (in Chinese)

4. Tieqiang Wang, Qingming Zheng, Yunjie Wu, Yunying Yao, Cuiping Kuang, Li X: **Investigation of economic burden caused by varicella in school aged children in Shenzhen**. *Disease Surveillance* 2021, **36**(10):1092-1095. (in Chinese)

5. Zhengbo Tu, Duan Li, Nie P: **Analysis of the Epidemiological Characteristics and Economic Burden of Other Infectious Diarrhea Disease in Honggutan District of Nanchang City From 2014 to 2016**. *Journal of Preventive Medicine Information* 2018, **34**(6):728-732. (in Chinese)

6. Zheng Y, Jit M, Wu JT, Yang J, Leung K, Liao Q *et al*: **Economic costs and health-related quality of life for hand, foot and mouth disease (HFMD) patients in China**. *Plos One* 2017, **12**(9):e0184266.
